# Supplementary material for: Acute Muscle Rigidity Secondary to Tetanus: A Toxicology Simulation Case for Fourth-Year Medical Students
Source: MedEdPORTAL. 2024 Mar 29;20:11389. doi: 10.15766/mep_2374-8265.11389 (PMC10978813; doi:10.15766/mep_2374-8265.11389)
Supplement: Supplementary file 1 — Approach to Acid-Base Disturbances.pptxGlycine.pptxSimulation Images and Lab Values.docxSimulation Case.docxCritical Actions Checklist.docxDebriefing Materials.docxPre- and Posttest.docxSession Evaluation.docx [file mep_2374-8265.11389-s001.zip › H. Session Evaluation.docx]

**Appendix H: Session evaluation**

Instructions: Following the simulation and debrief, use this resource to anonymously allow learners to evaluate the simulation session. We recommend allowing learners 5 minutes to complete this.

Please respond with a number between 1-5 which correspond to the following:

5: Strongly agree

4: Agree

3: Neutral

2: Disagree

1: Strongly disagree

Statement: The “Glycine” lecture assisted my learning, Response:

Statement: The “Acid-Base” lecture assisted my learning, Response:

Statement: The simulation content was relevant to my medical education, Response:

Statement: The simulation content was appropriate for my level of training, Response:

Statement: The simulation helped build inter-professional skills, Response:

Statement: The simulation should be continued, Response:

Statement: I feel more prepared to care for a patient with tetanus, Response:
