# Supplementary material for: Acute Muscle Rigidity Secondary to Tetanus: A Toxicology Simulation Case for Fourth-Year Medical Students
Source: MedEdPORTAL. 2024 Mar 29;20:11389. doi: 10.15766/mep_2374-8265.11389 (PMC10978813; doi:10.15766/mep_2374-8265.11389)
Supplement: Supplementary file 1 — Approach to Acid-Base Disturbances.pptxGlycine.pptxSimulation Images and Lab Values.docxSimulation Case.docxCritical Actions Checklist.docxDebriefing Materials.docxPre- and Posttest.docxSession Evaluation.docx [file mep_2374-8265.11389-s001.zip › A. Approach to Acid-Base Disturbances.pptx]

## Slide 1
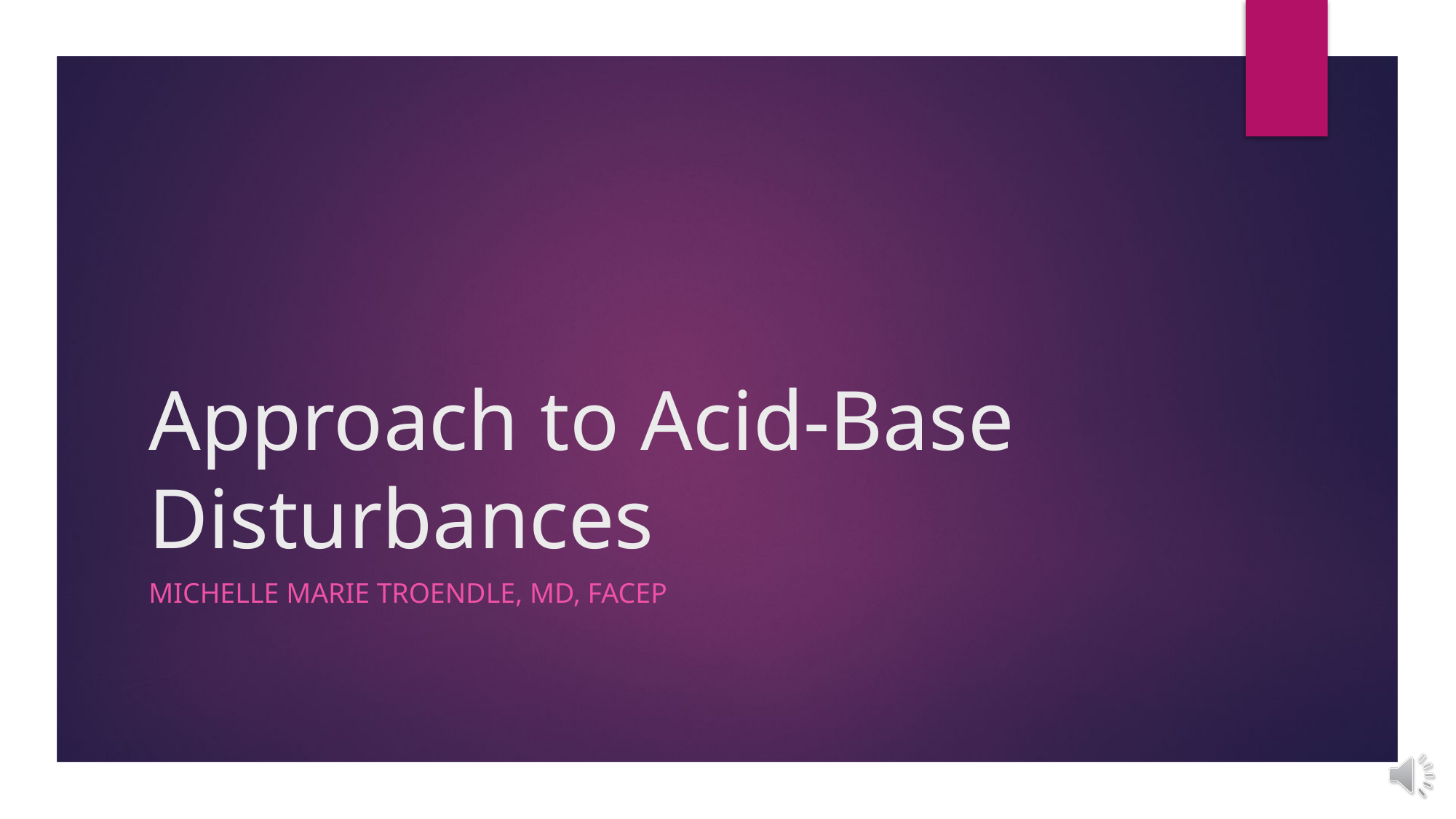

# Approach to Acid-Base Disturbances
MICHELLE MARIE TROENDLE, MD, FACEP

## Slide 2
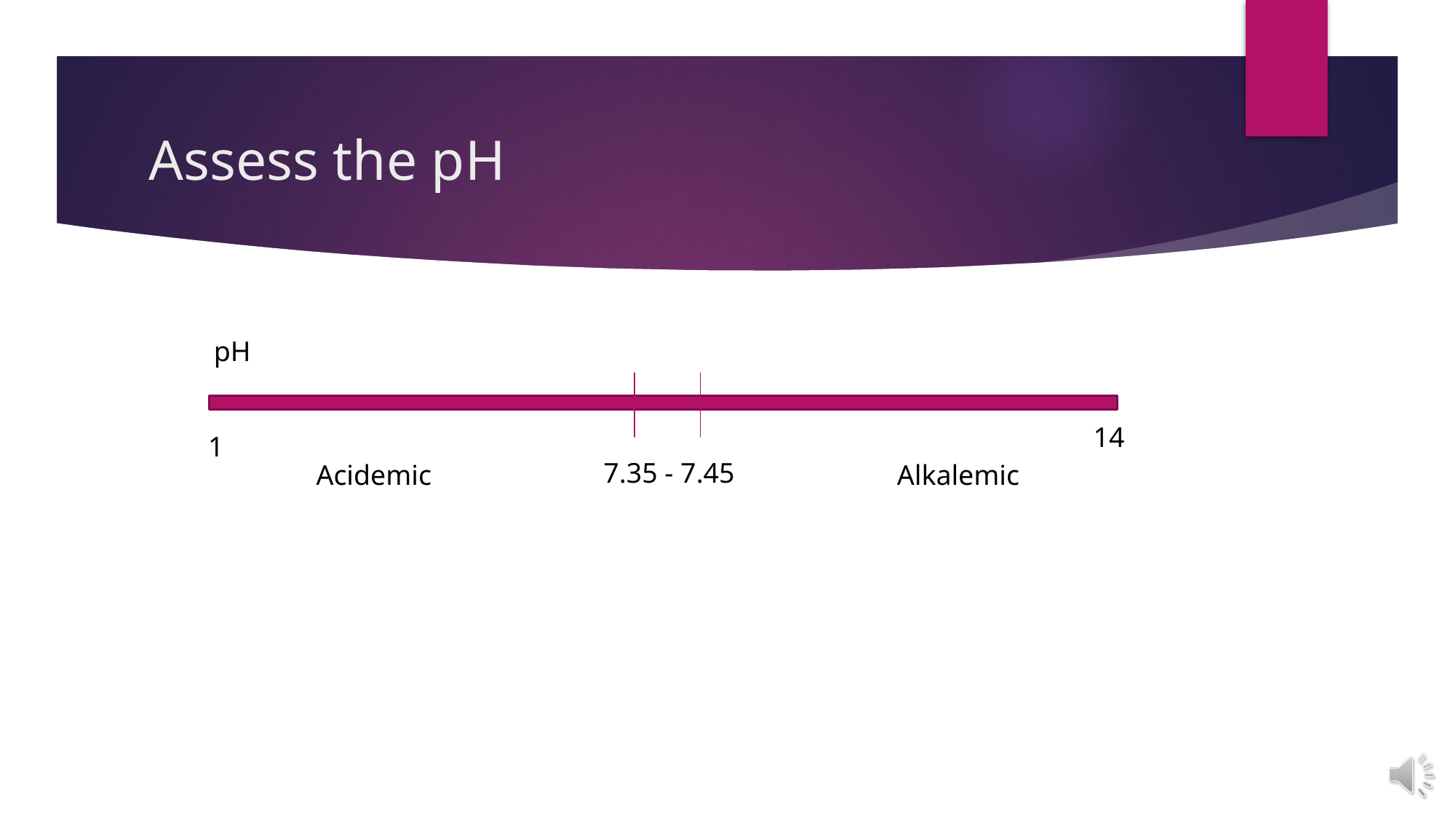

# Assess the pH
pH
14
1
7.35 - 7.45
Acidemic
Alkalemic

## Slide 3
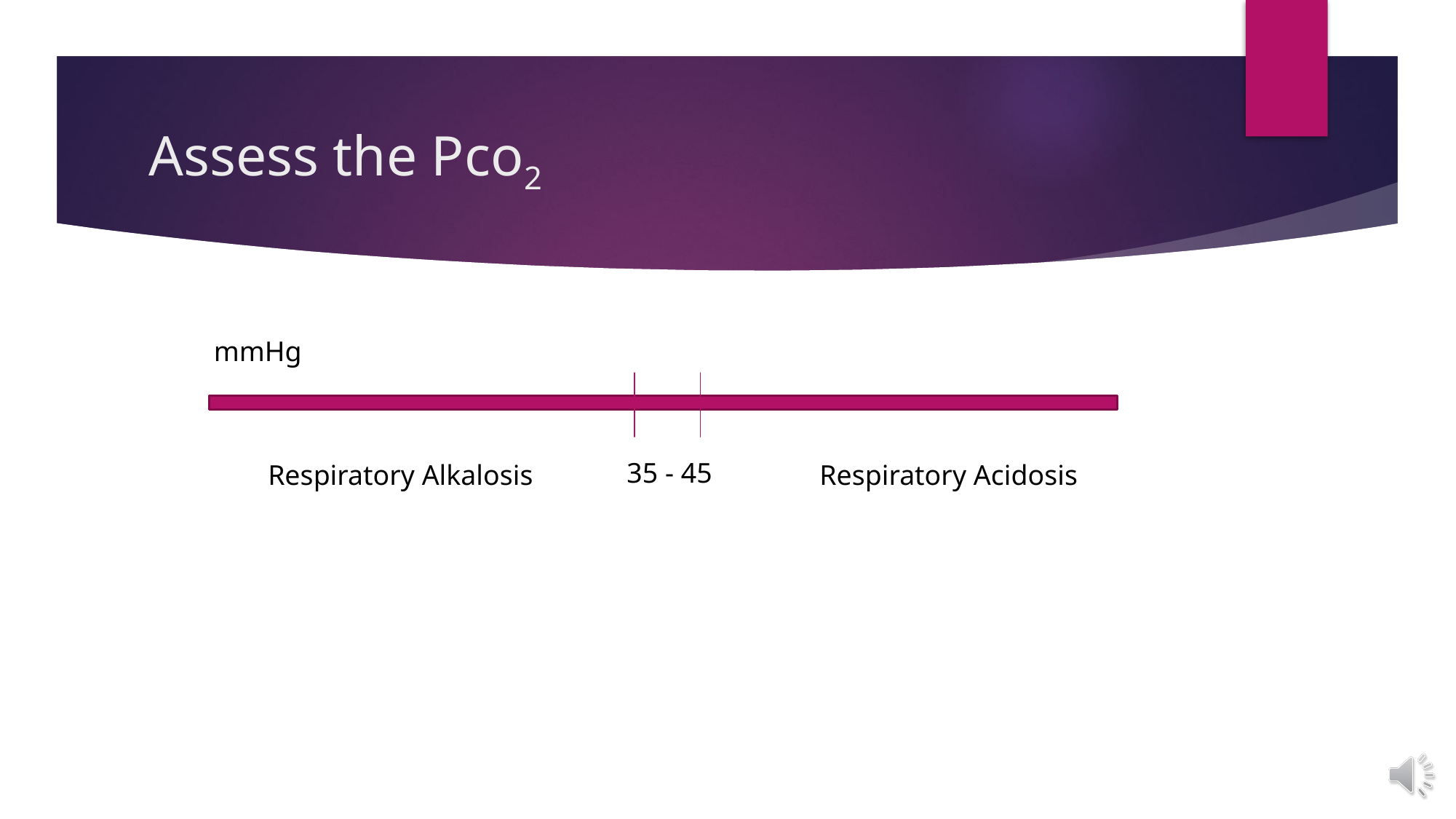

# Assess the Pco2
mmHg
35 - 45
Respiratory Alkalosis
Respiratory Acidosis

## Slide 4
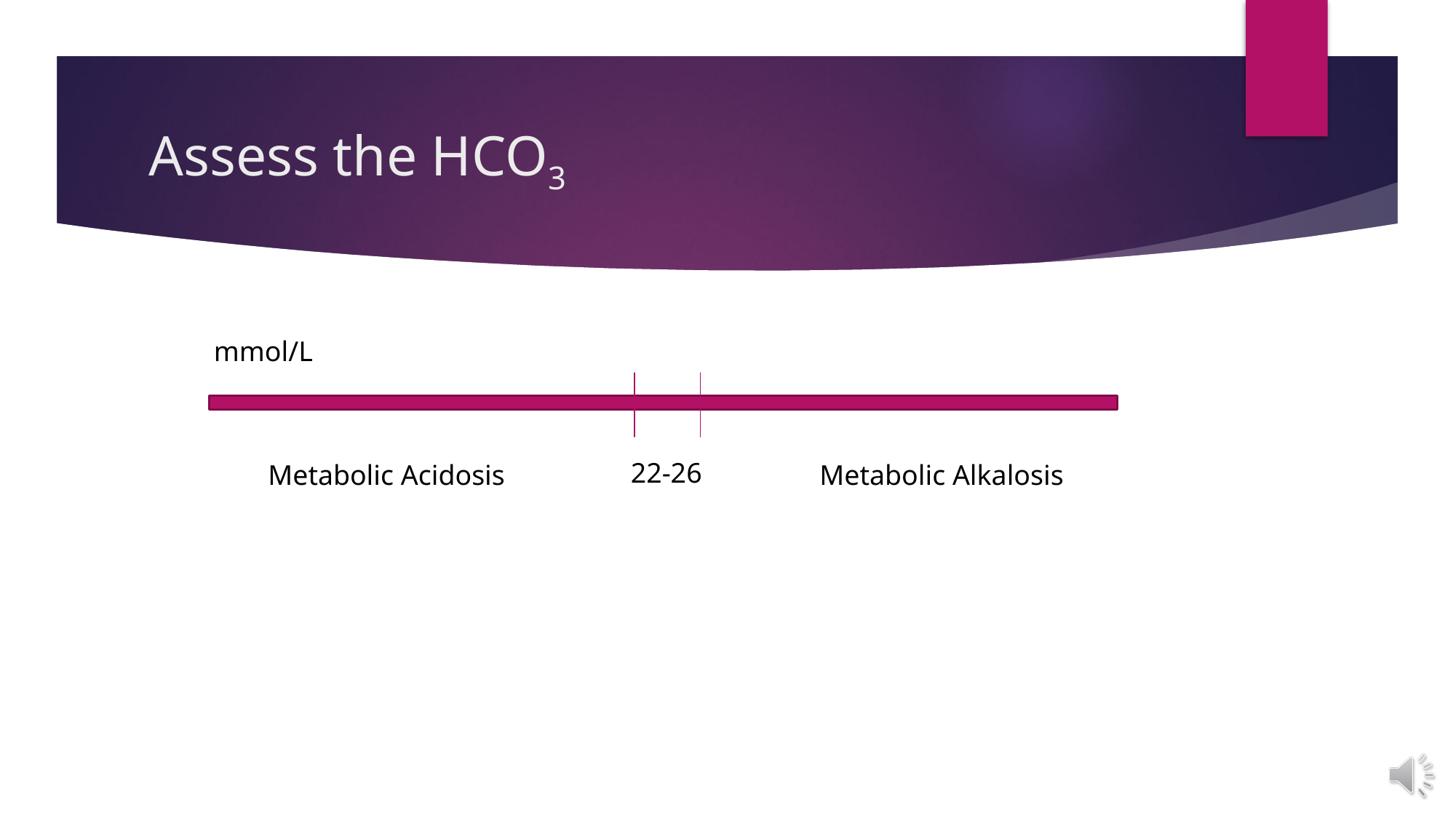

# Assess the HCO3
mmol/L
22-26
Metabolic Acidosis
Metabolic Alkalosis

## Slide 5
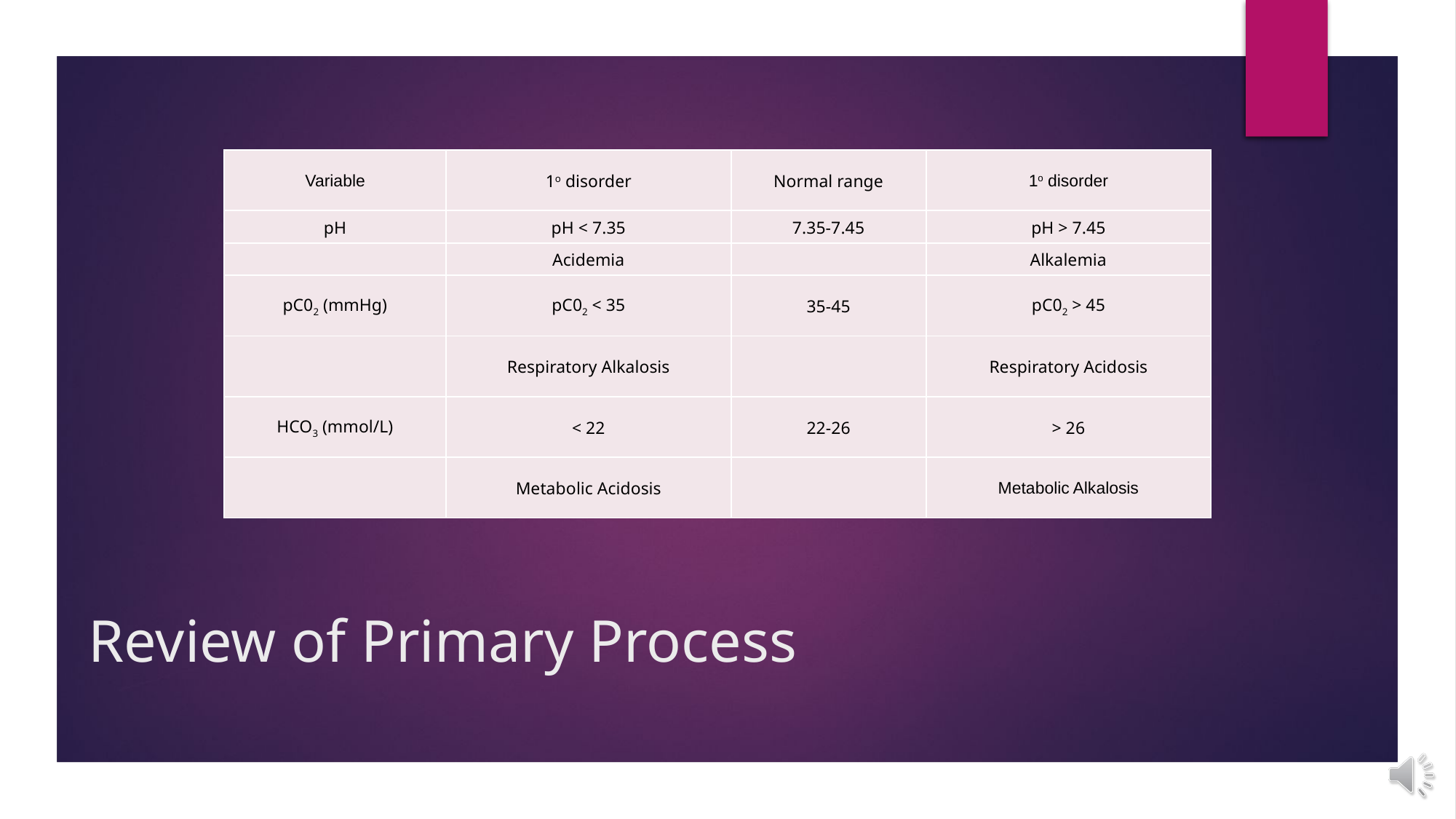

| Variable | 1o disorder | Normal range | 1o disorder |
| --- | --- | --- | --- |
| pH | pH < 7.35 | 7.35-7.45 | pH > 7.45 |
| | Acidemia | | Alkalemia |
| pC02 (mmHg) | pC02 < 35 | 35-45 | pC02 > 45 |
| | Respiratory Alkalosis | | Respiratory Acidosis |
| HCO3 (mmol/L) | < 22 | 22-26 | > 26 |
| | Metabolic Acidosis | | Metabolic Alkalosis |
# Review of Primary Process

## Slide 6
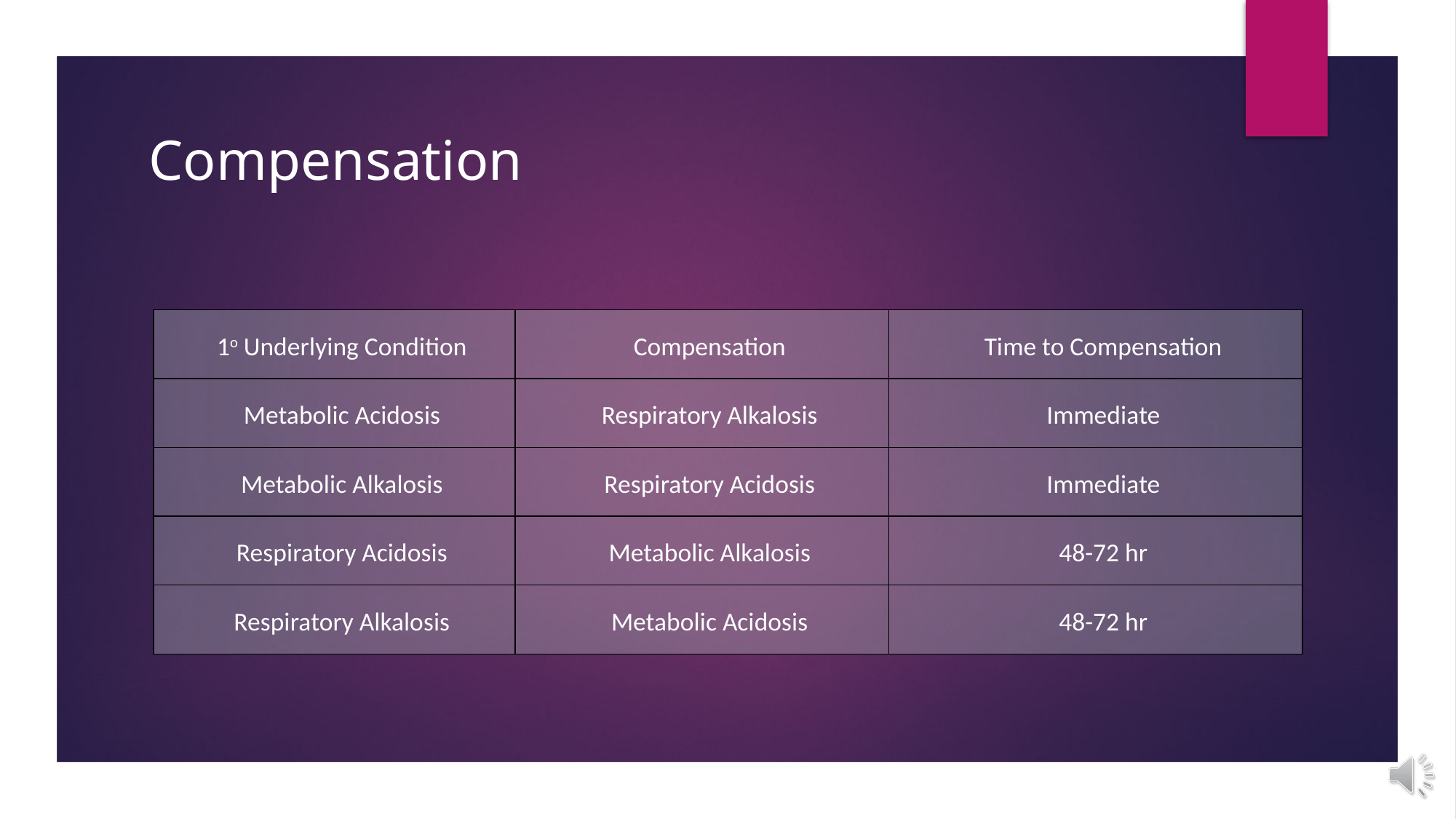

# Compensation
| 1o Underlying Condition | Compensation | Time to Compensation |
| --- | --- | --- |
| Metabolic Acidosis | Respiratory Alkalosis | Immediate |
| Metabolic Alkalosis | Respiratory Acidosis | Immediate |
| Respiratory Acidosis | Metabolic Alkalosis | 48-72 hr |
| Respiratory Alkalosis | Metabolic Acidosis | 48-72 hr |

## Slide 7
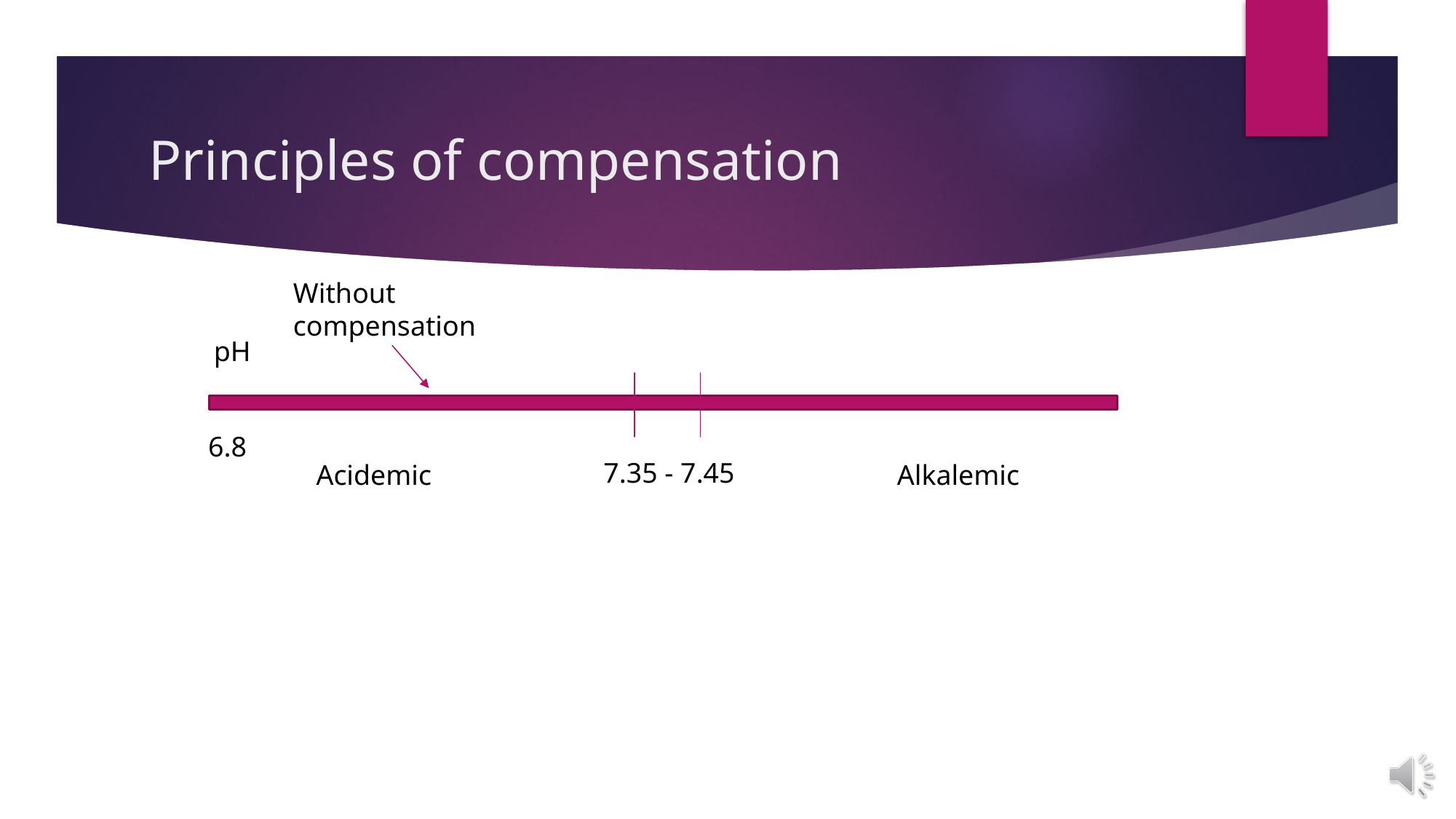

# Principles of compensation
Without compensation
pH
6.8
7.35 - 7.45
Acidemic
Alkalemic

## Slide 8
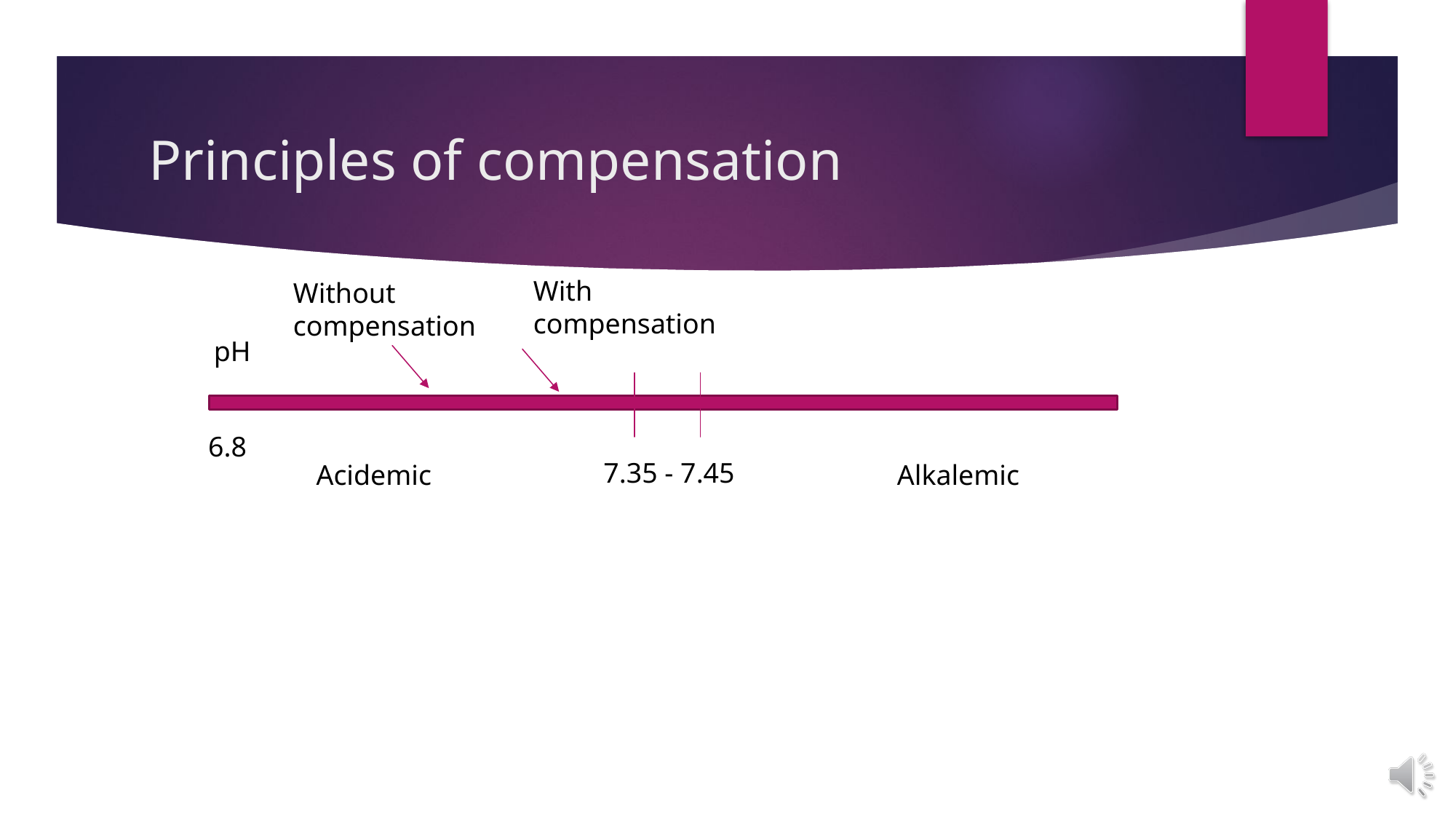

# Principles of compensation
With compensation
Without compensation
pH
6.8
7.35 - 7.45
Acidemic
Alkalemic

## Slide 9
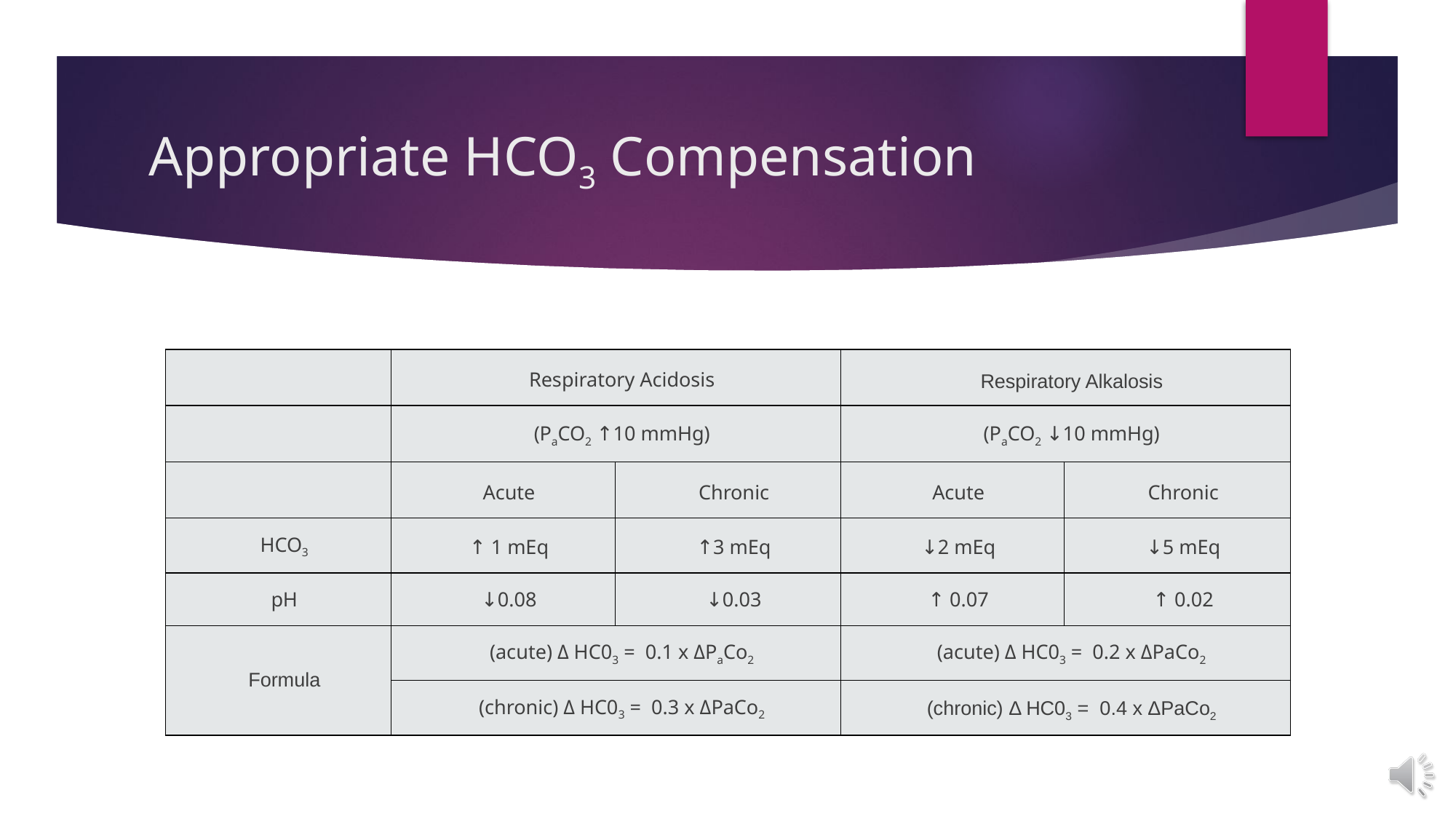

# Appropriate HCO3 Compensation
| | Respiratory Acidosis | | Respiratory Alkalosis | |
| --- | --- | --- | --- | --- |
| | (PaCO2 ↑10 mmHg) | | (PaCO2 ↓10 mmHg) | |
| | Acute | Chronic | Acute | Chronic |
| HCO3 | ↑ 1 mEq | ↑3 mEq | ↓2 mEq | ↓5 mEq |
| pH | ↓0.08 | ↓0.03 | ↑ 0.07 | ↑ 0.02 |
| Formula | (acute) Δ HC03 = 0.1 x ΔPaCo2 | | (acute) Δ HC03 = 0.2 x ΔPaCo2 | |
| | (chronic) Δ HC03 = 0.3 x ΔPaCo2 | | (chronic) Δ HC03 = 0.4 x ΔPaCo2 | |

## Slide 10
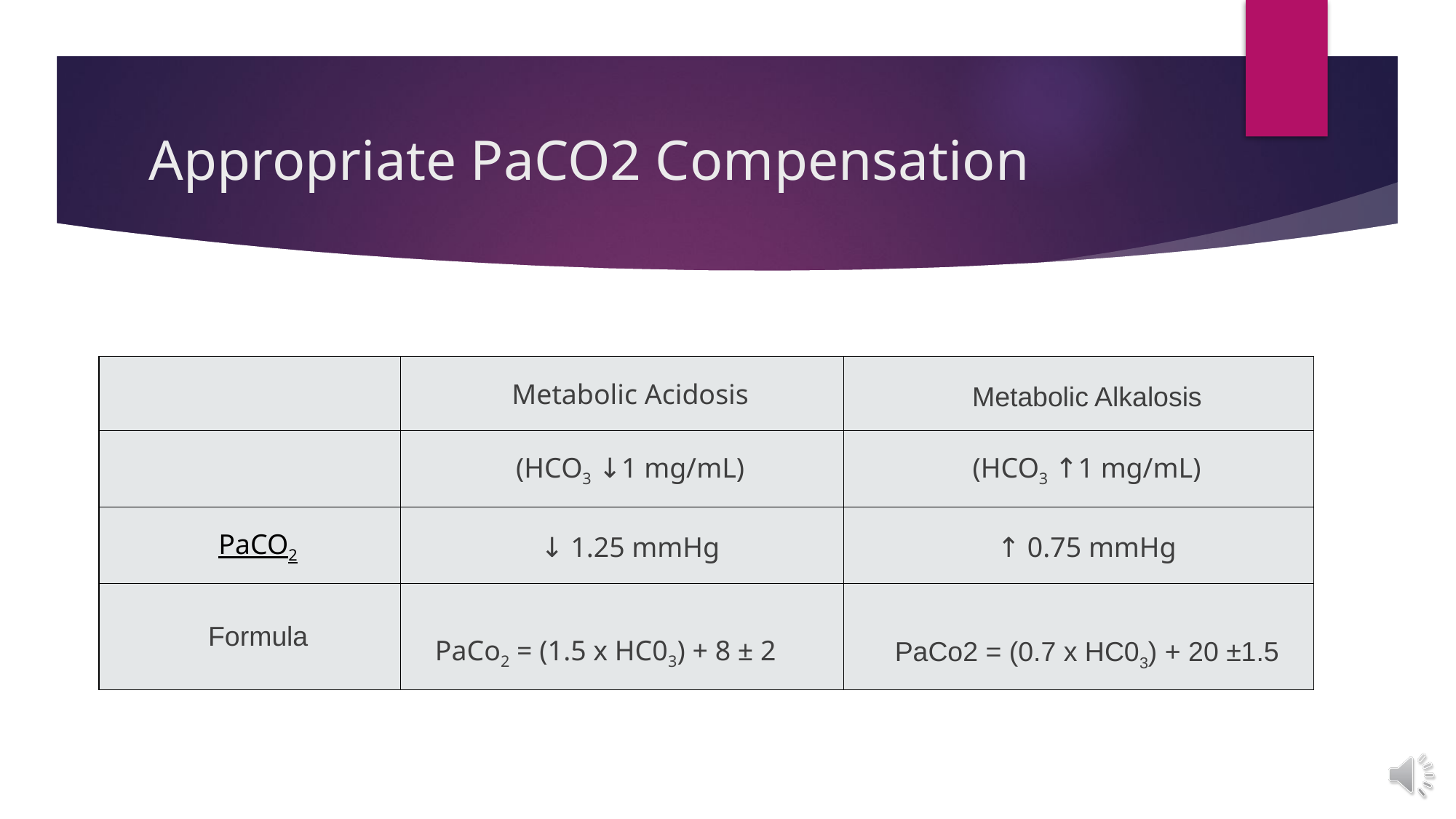

# Appropriate PaCO2 Compensation
| | Metabolic Acidosis | Metabolic Alkalosis |
| --- | --- | --- |
| | (HCO3 ↓1 mg/mL) | (HCO3 ↑1 mg/mL) |
| PaCO2 | ↓ 1.25 mmHg | ↑ 0.75 mmHg |
| Formula | PaCo2 = (1.5 x HC03) + 8 ± 2 | PaCo2 = (0.7 x HC03) + 20 ±1.5 |

## Slide 11
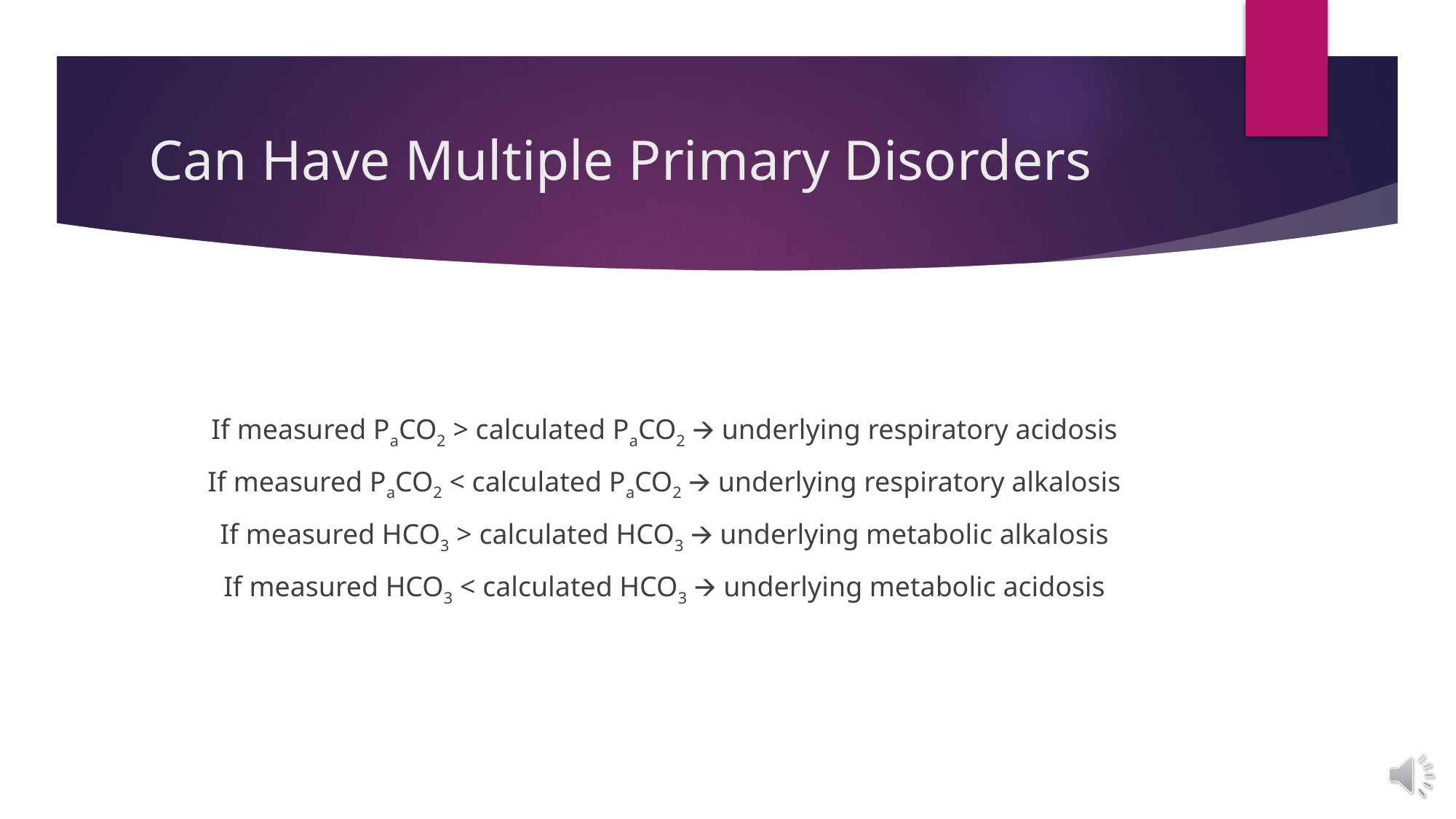

# Can Have Multiple Primary Disorders
If measured PaCO2 > calculated PaCO2 🡪 underlying respiratory acidosis
If measured PaCO2 < calculated PaCO2 🡪 underlying respiratory alkalosis
If measured HCO3 > calculated HCO3 🡪 underlying metabolic alkalosis
If measured HCO3 < calculated HCO3 🡪 underlying metabolic acidosis

## Slide 12
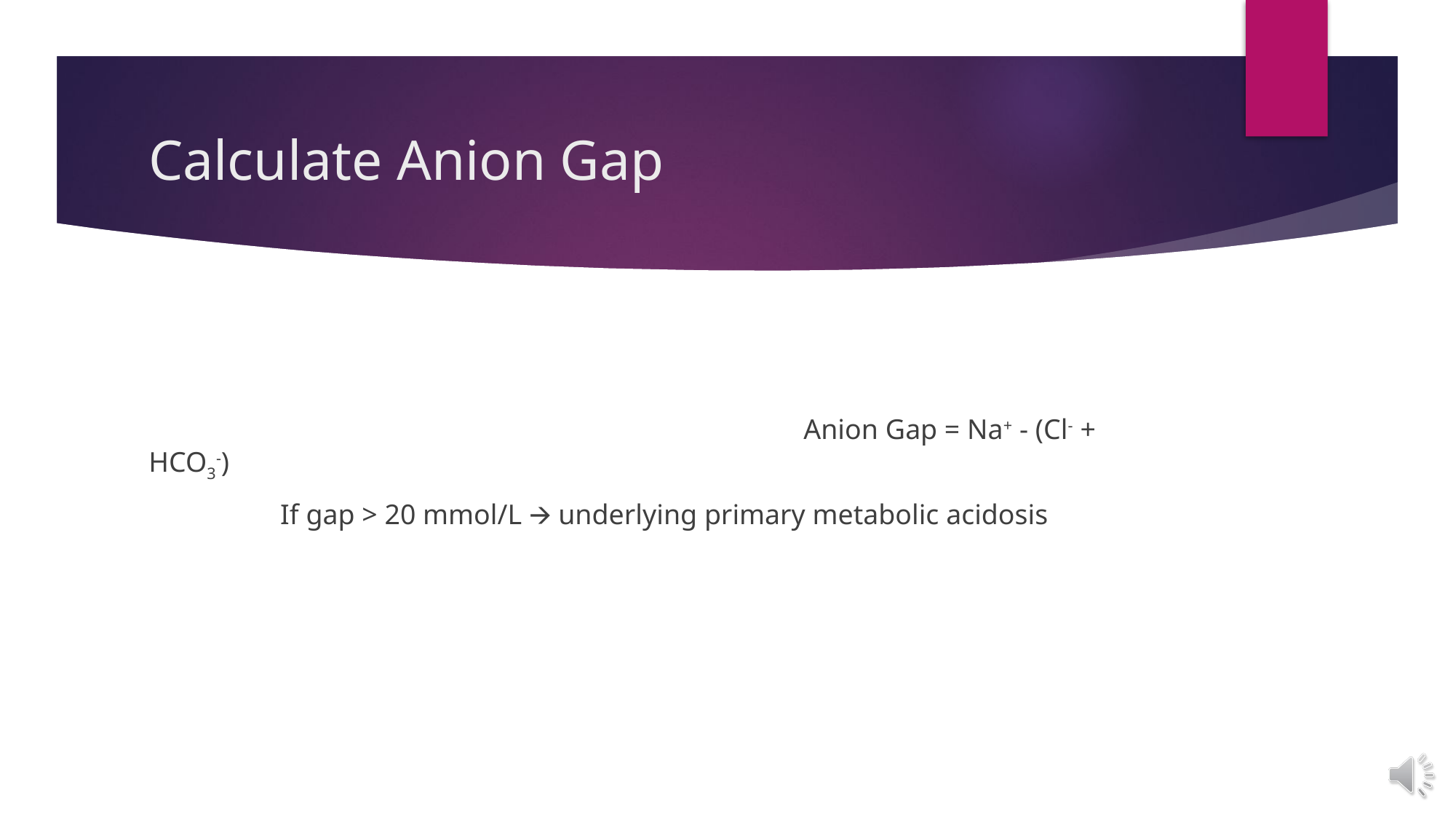

# Calculate Anion Gap
						Anion Gap = Na+ - (Cl- + HCO3-)
If gap > 20 mmol/L 🡪 underlying primary metabolic acidosis

## Slide 13
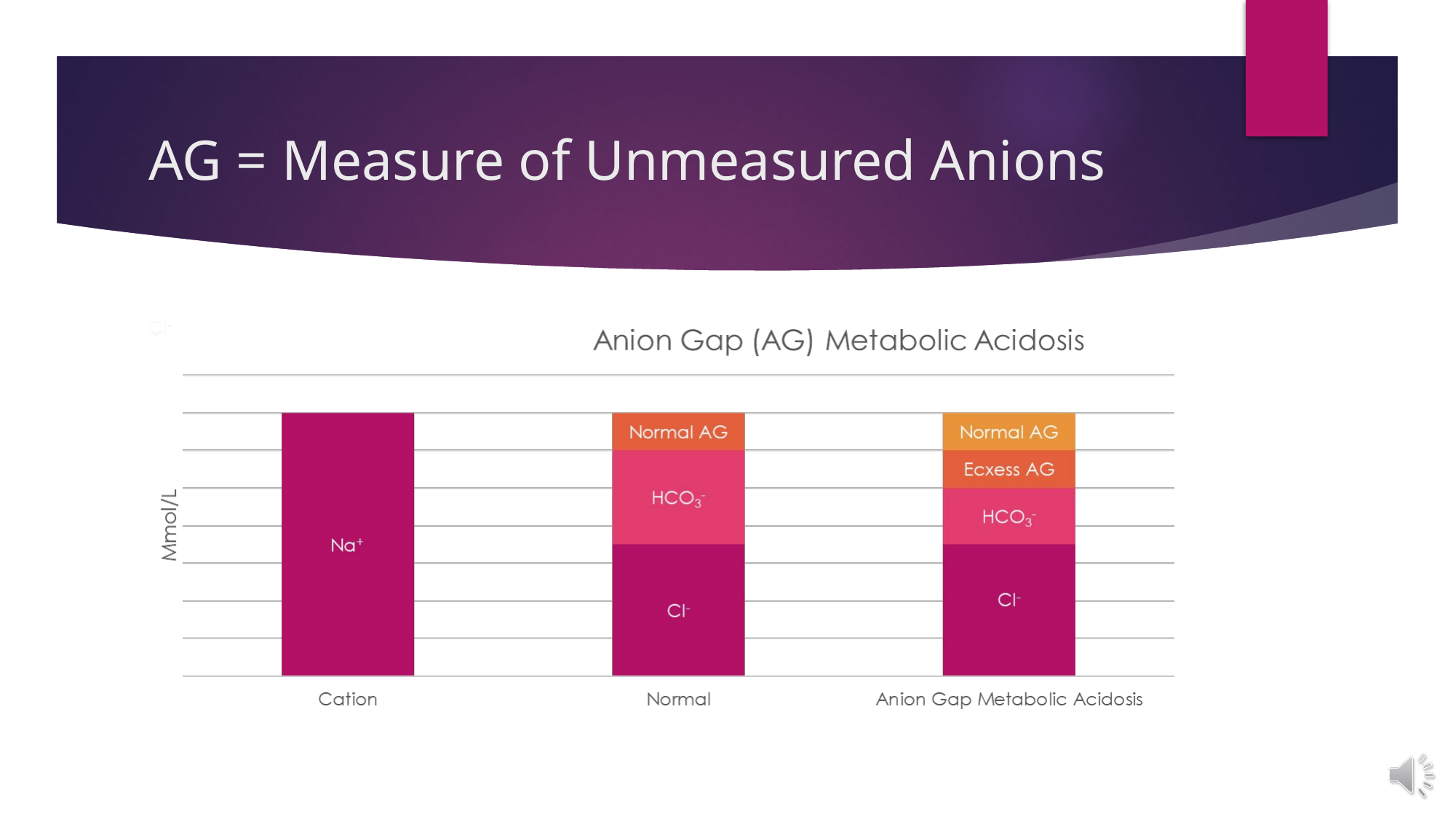

# AG = Measure of Unmeasured Anions

## Slide 14
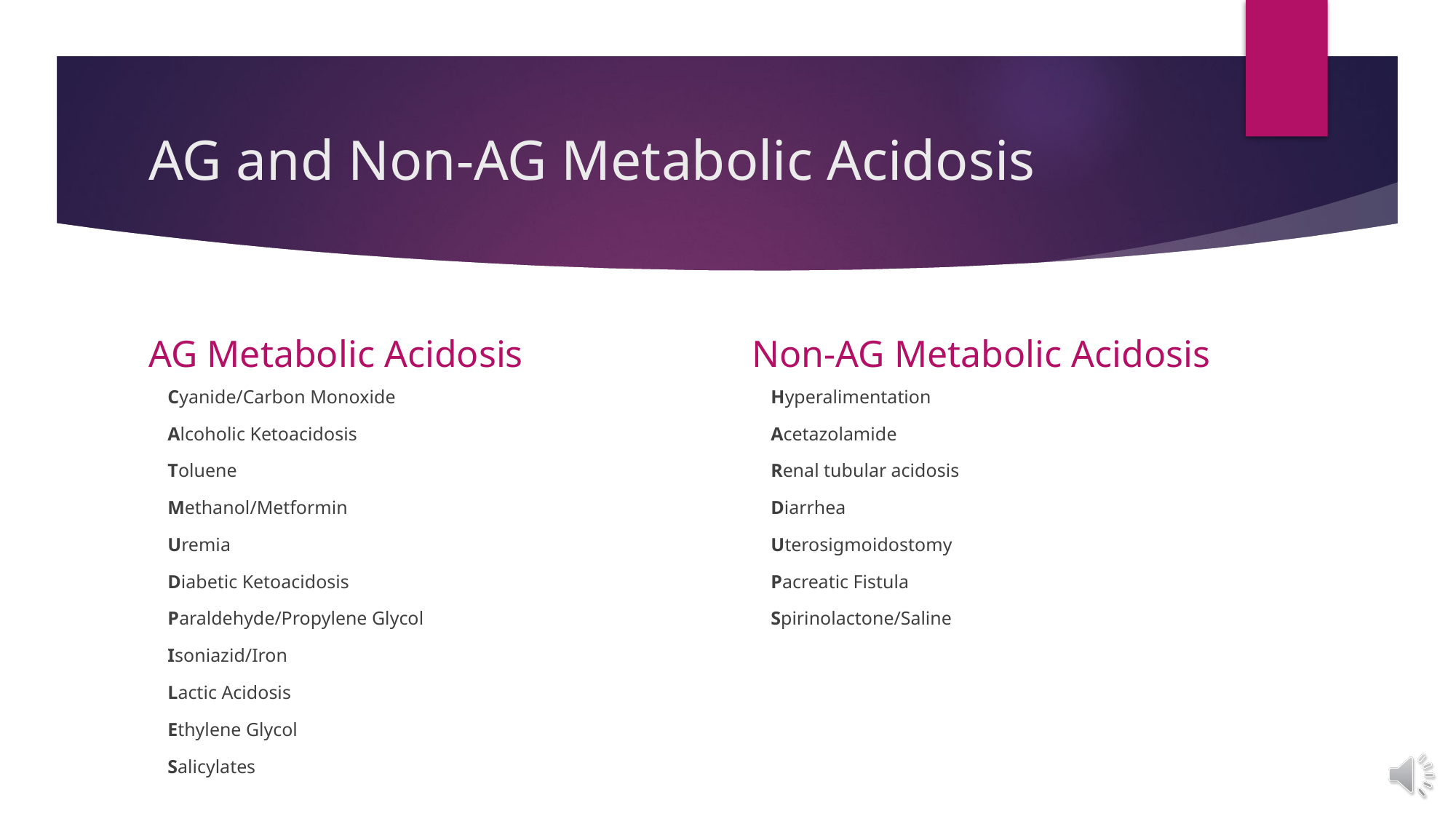

# AG and Non-AG Metabolic Acidosis
AG Metabolic Acidosis
Non-AG Metabolic Acidosis
 Cyanide/Carbon Monoxide
 Alcoholic Ketoacidosis
 Toluene
 Methanol/Metformin
 Uremia
 Diabetic Ketoacidosis
 Paraldehyde/Propylene Glycol
 Isoniazid/Iron
 Lactic Acidosis
 Ethylene Glycol
 Salicylates
 Hyperalimentation
 Acetazolamide
 Renal tubular acidosis
 Diarrhea
 Uterosigmoidostomy
 Pacreatic Fistula
 Spirinolactone/Saline

## Slide 15
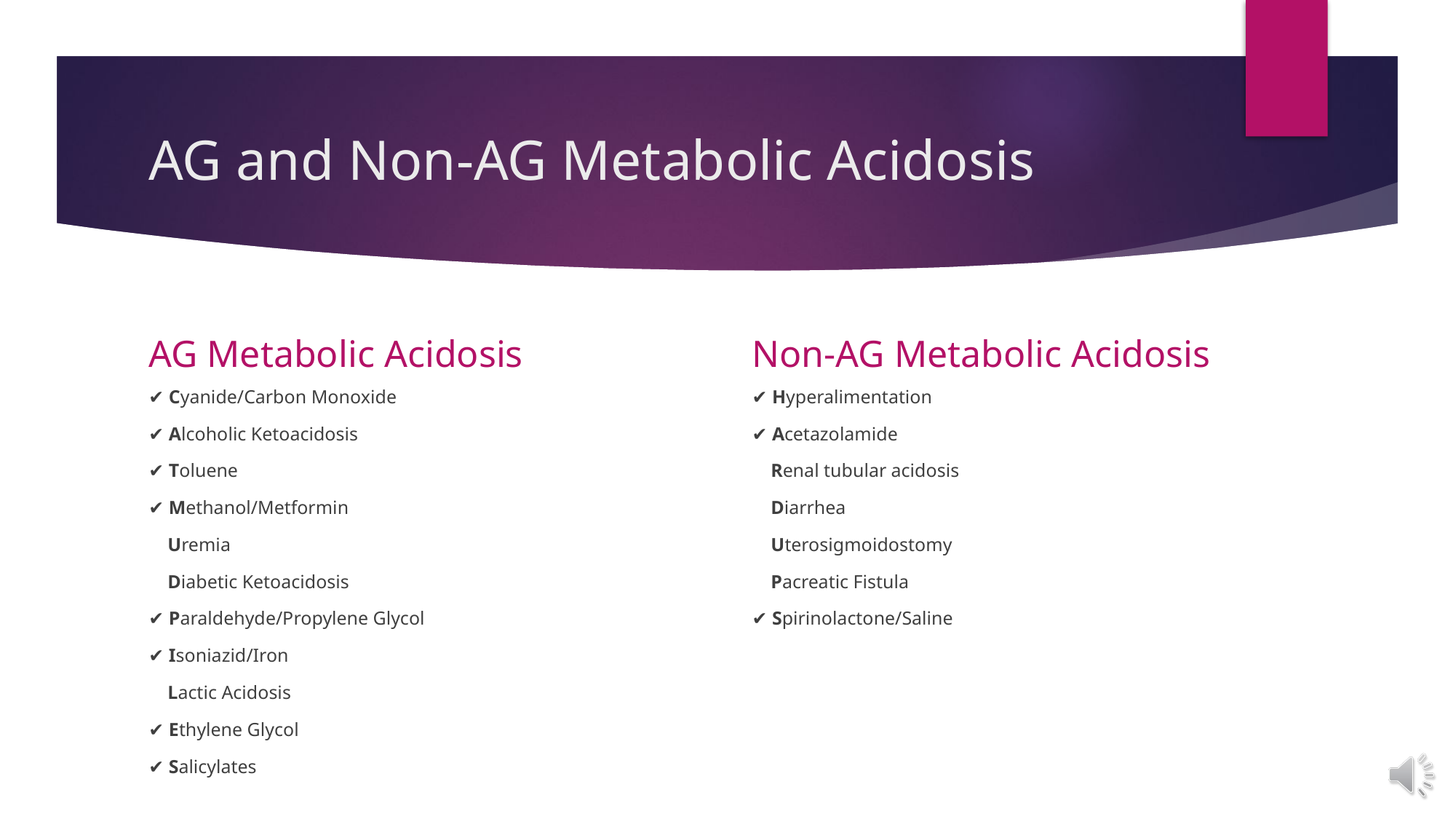

# AG and Non-AG Metabolic Acidosis
AG Metabolic Acidosis
Non-AG Metabolic Acidosis
✔ Cyanide/Carbon Monoxide
✔ Alcoholic Ketoacidosis
✔ Toluene
✔ Methanol/Metformin
 Uremia
 Diabetic Ketoacidosis
✔ Paraldehyde/Propylene Glycol
✔ Isoniazid/Iron
 Lactic Acidosis
✔ Ethylene Glycol
✔ Salicylates
✔ Hyperalimentation
✔ Acetazolamide
 Renal tubular acidosis
 Diarrhea
 Uterosigmoidostomy
 Pacreatic Fistula
✔ Spirinolactone/Saline

## Slide 16
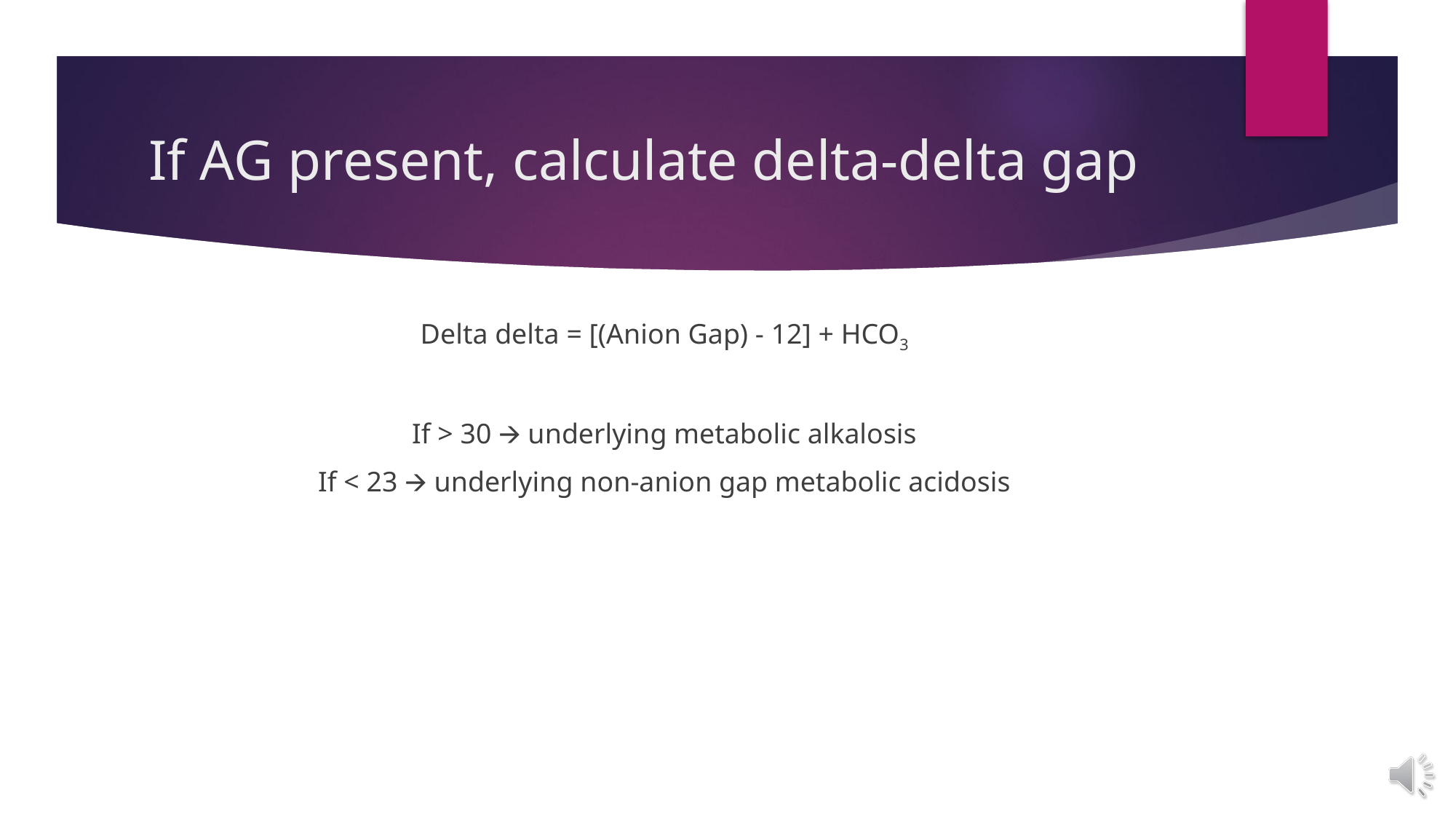

# If AG present, calculate delta-delta gap
Delta delta = [(Anion Gap) - 12] + HCO3
If > 30 🡪 underlying metabolic alkalosis
If < 23 🡪 underlying non-anion gap metabolic acidosis

## Slide 17
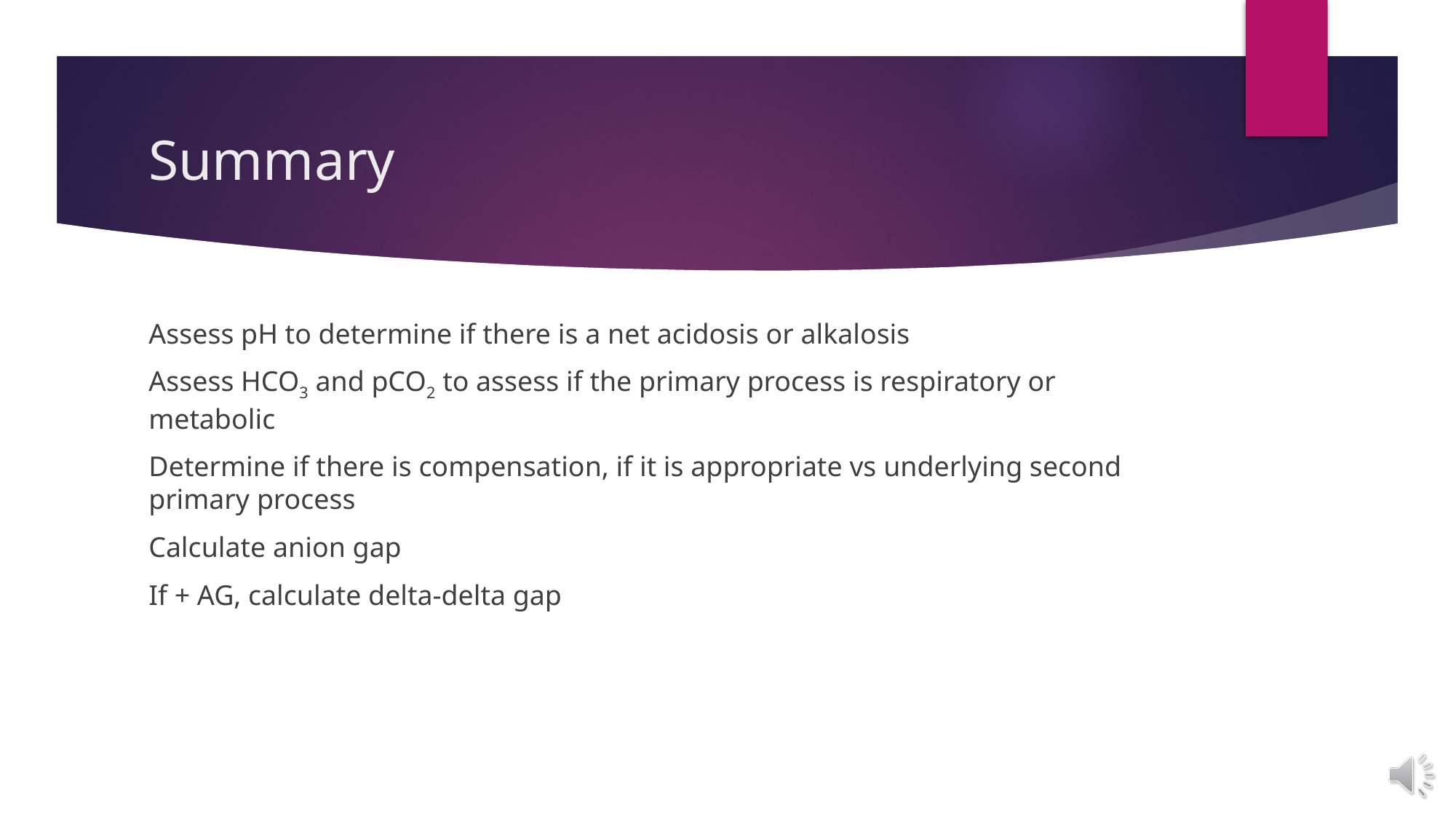

# Summary
Assess pH to determine if there is a net acidosis or alkalosis
Assess HCO3 and pCO2 to assess if the primary process is respiratory or metabolic
Determine if there is compensation, if it is appropriate vs underlying second primary process
Calculate anion gap
If + AG, calculate delta-delta gap

## Slide 18
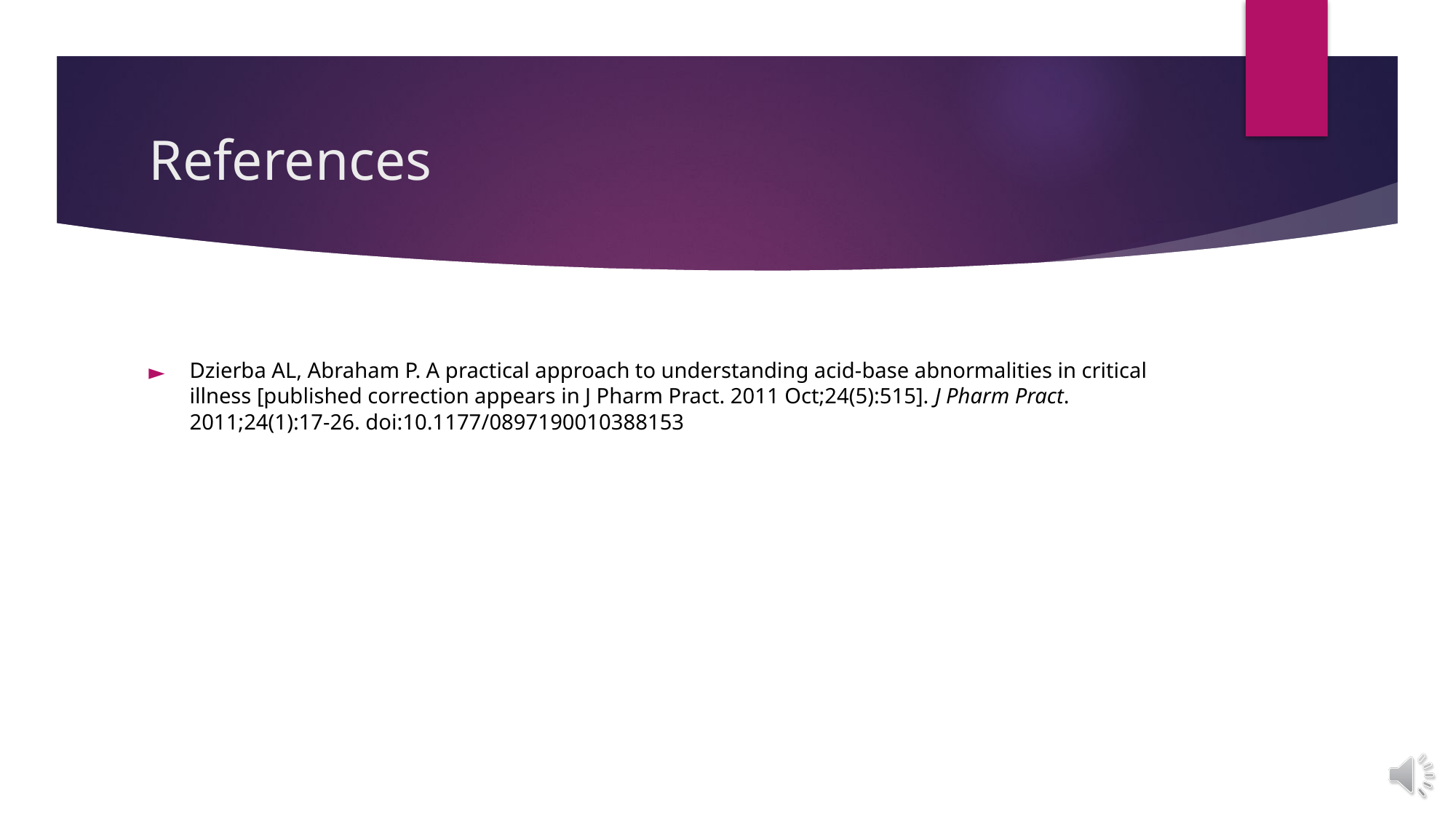

# References
Dzierba AL, Abraham P. A practical approach to understanding acid-base abnormalities in critical illness [published correction appears in J Pharm Pract. 2011 Oct;24(5):515]. J Pharm Pract. 2011;24(1):17-26. doi:10.1177/0897190010388153
