# Supplementary material for: Acute Muscle Rigidity Secondary to Tetanus: A Toxicology Simulation Case for Fourth-Year Medical Students
Source: MedEdPORTAL. 2024 Mar 29;20:11389. doi: 10.15766/mep_2374-8265.11389 (PMC10978813; doi:10.15766/mep_2374-8265.11389)
Supplement: Supplementary file 1 — Approach to Acid-Base Disturbances.pptxGlycine.pptxSimulation Images and Lab Values.docxSimulation Case.docxCritical Actions Checklist.docxDebriefing Materials.docxPre- and Posttest.docxSession Evaluation.docx [file mep_2374-8265.11389-s001.zip › B. Glycine.pptx]

## Slide 1
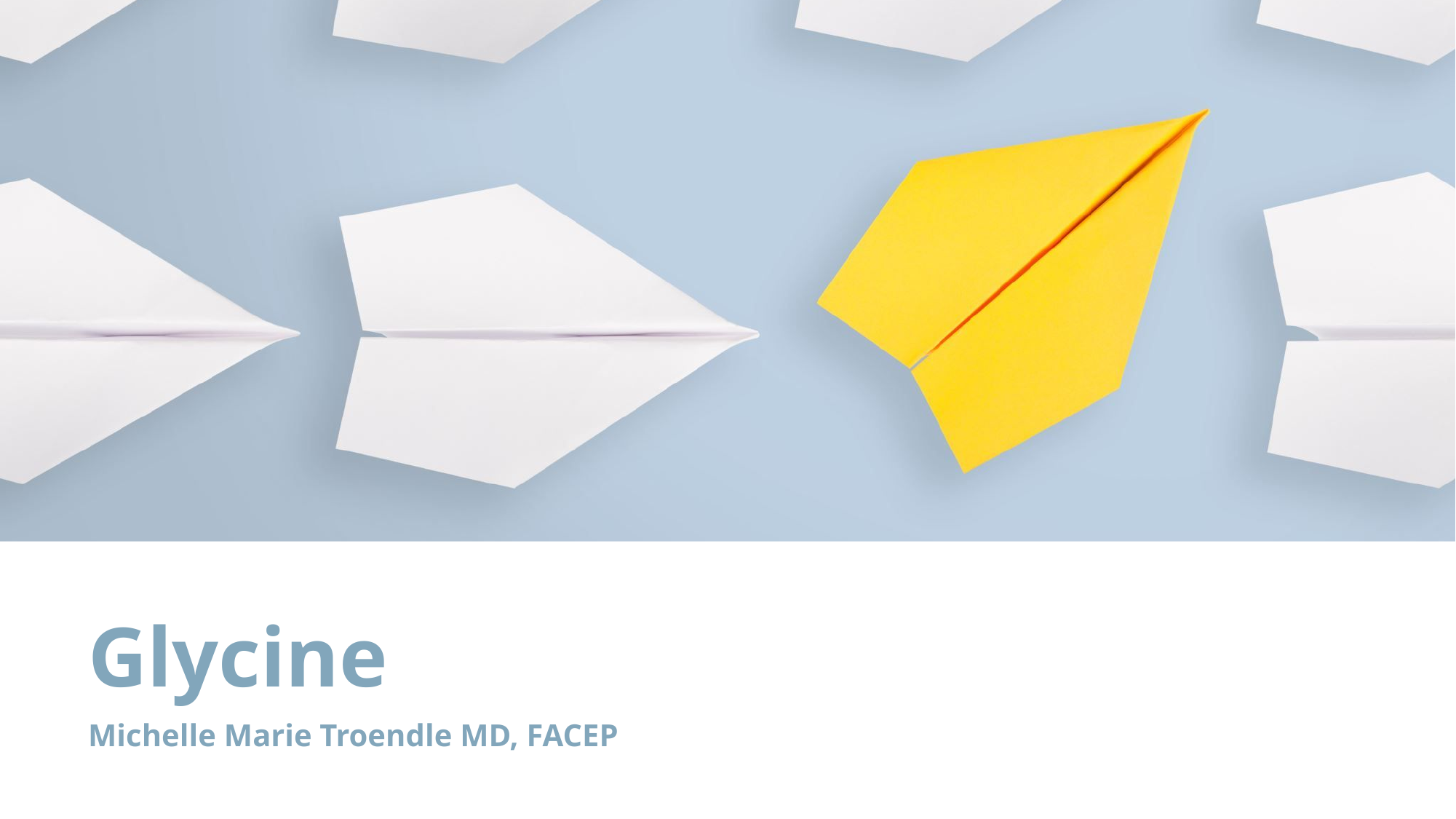

# Glycine
Michelle Marie Troendle MD, FACEP

## Slide 2
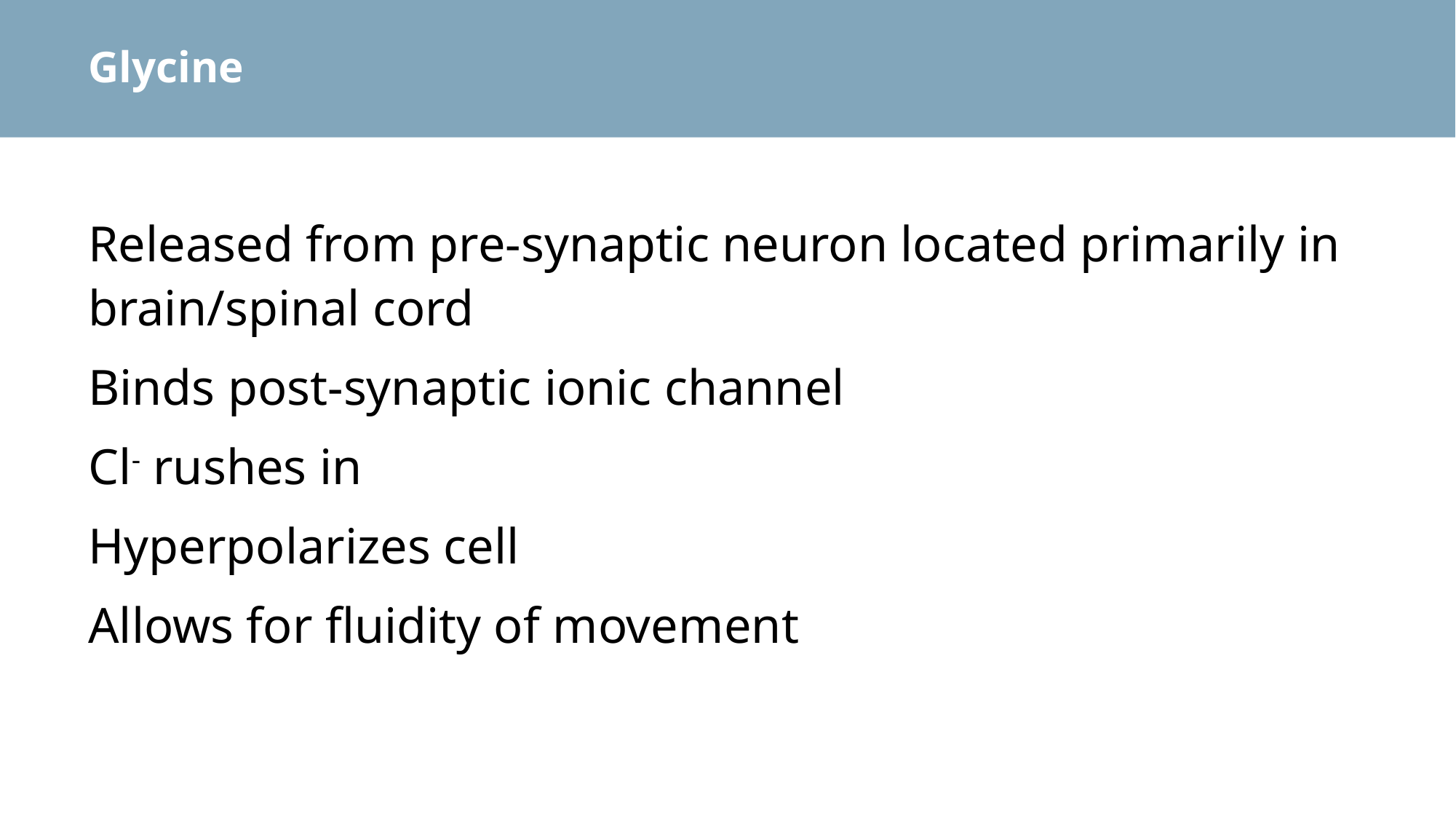

# Glycine
Released from pre-synaptic neuron located primarily in brain/spinal cord
Binds post-synaptic ionic channel
Cl- rushes in
Hyperpolarizes cell
Allows for fluidity of movement

## Slide 3
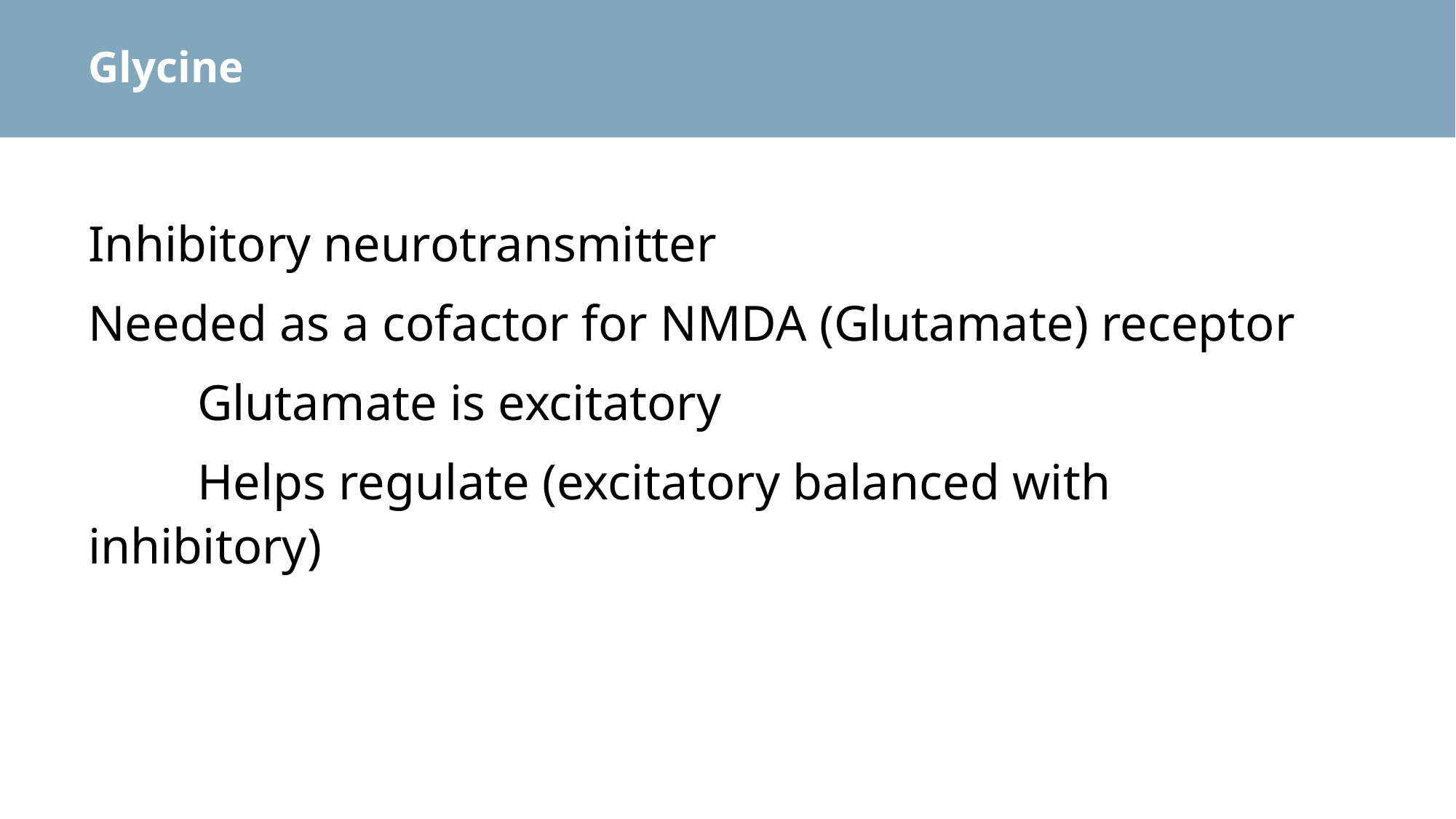

# Glycine
Inhibitory neurotransmitter
Needed as a cofactor for NMDA (Glutamate) receptor
	Glutamate is excitatory
	Helps regulate (excitatory balanced with inhibitory)

## Slide 4
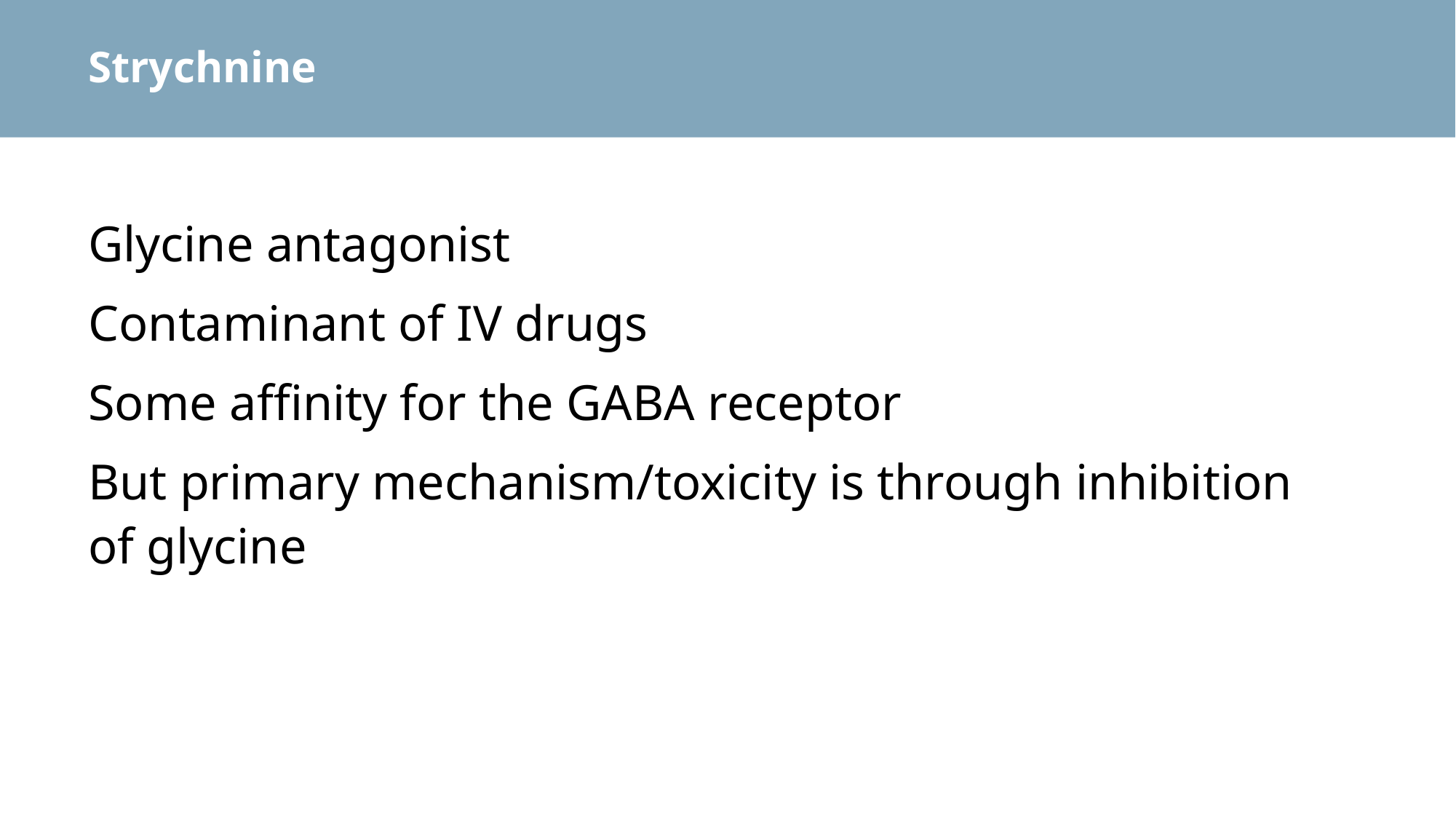

# Strychnine
Glycine antagonist
Contaminant of IV drugs
Some affinity for the GABA receptor
But primary mechanism/toxicity is through inhibition of glycine

## Slide 5
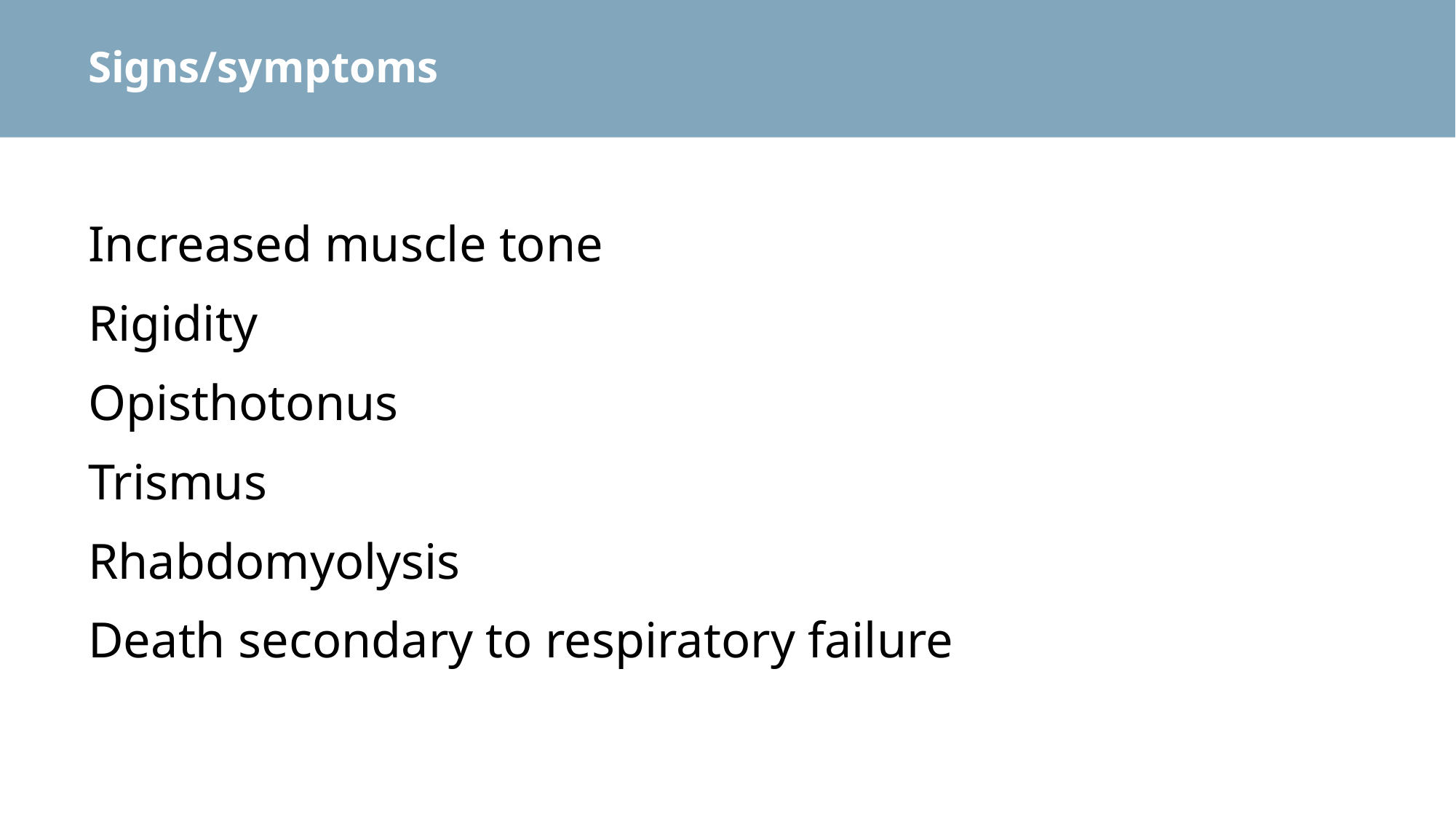

# Signs/symptoms
Increased muscle tone
Rigidity
Opisthotonus
Trismus
Rhabdomyolysis
Death secondary to respiratory failure

## Slide 6
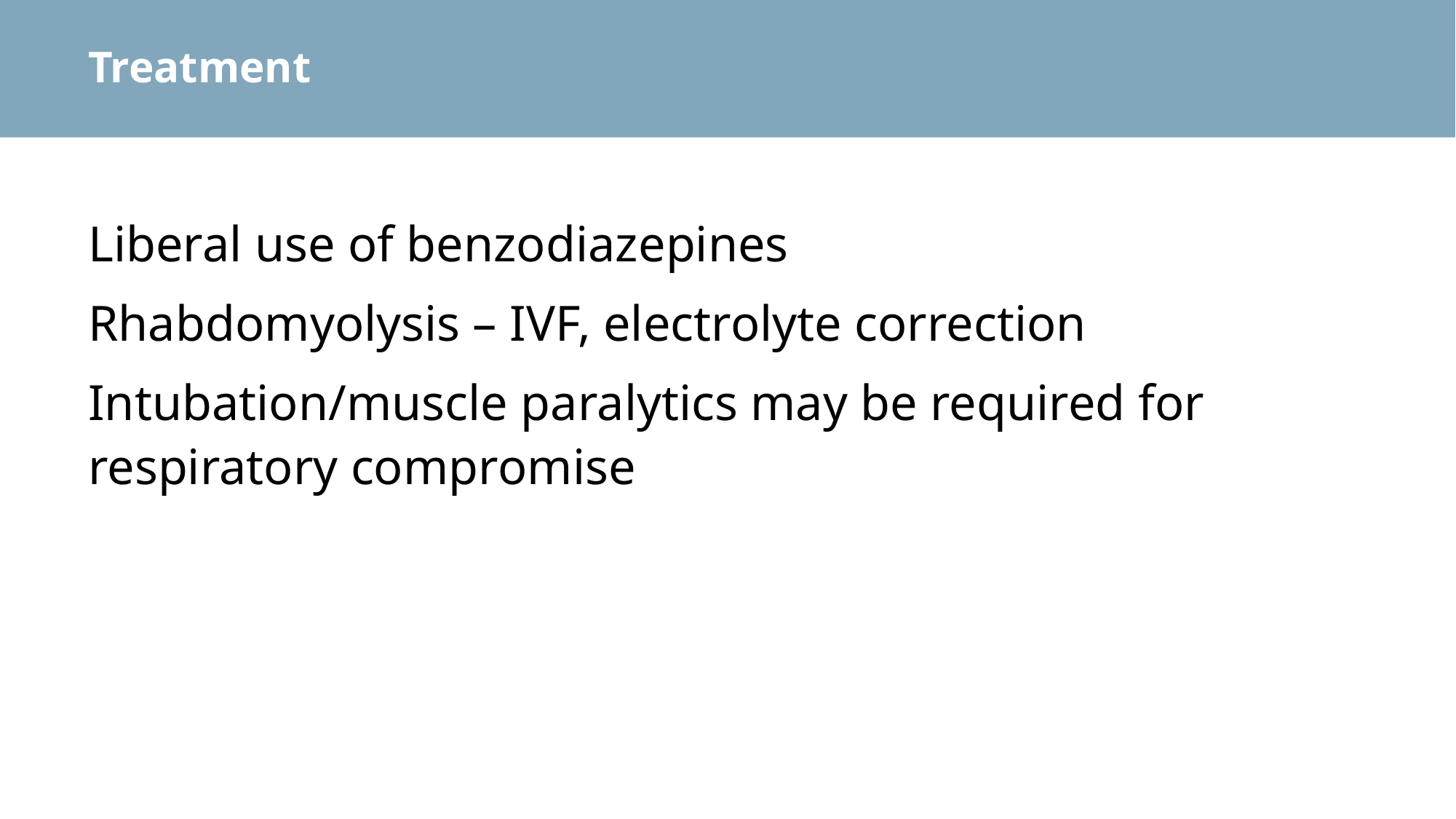

# Treatment
Liberal use of benzodiazepines
Rhabdomyolysis – IVF, electrolyte correction
Intubation/muscle paralytics may be required for respiratory compromise

## Slide 7
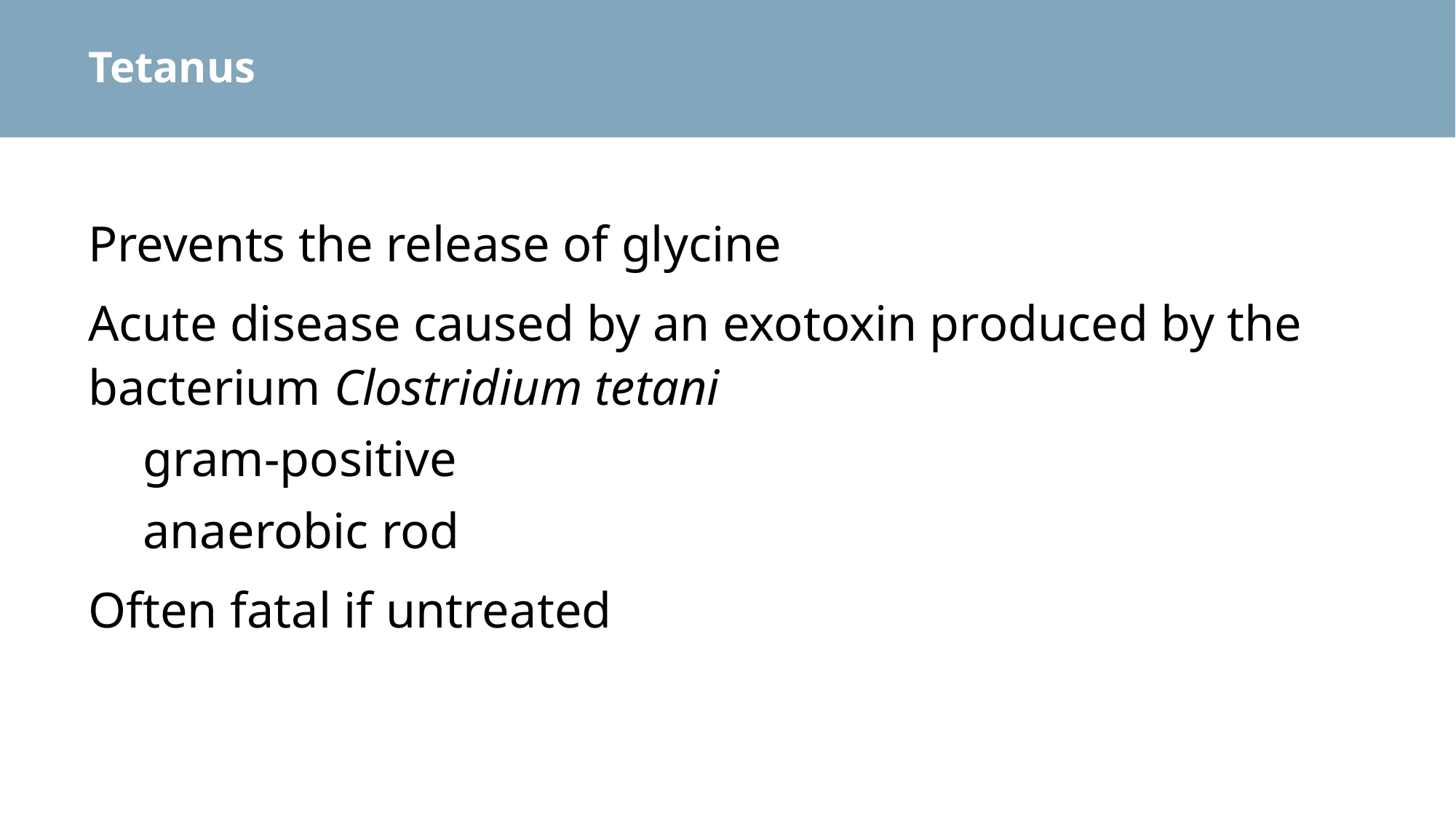

# Tetanus
Prevents the release of glycine
Acute disease caused by an exotoxin produced by the bacterium Clostridium tetani
gram-positive
anaerobic rod
Often fatal if untreated

## Slide 8
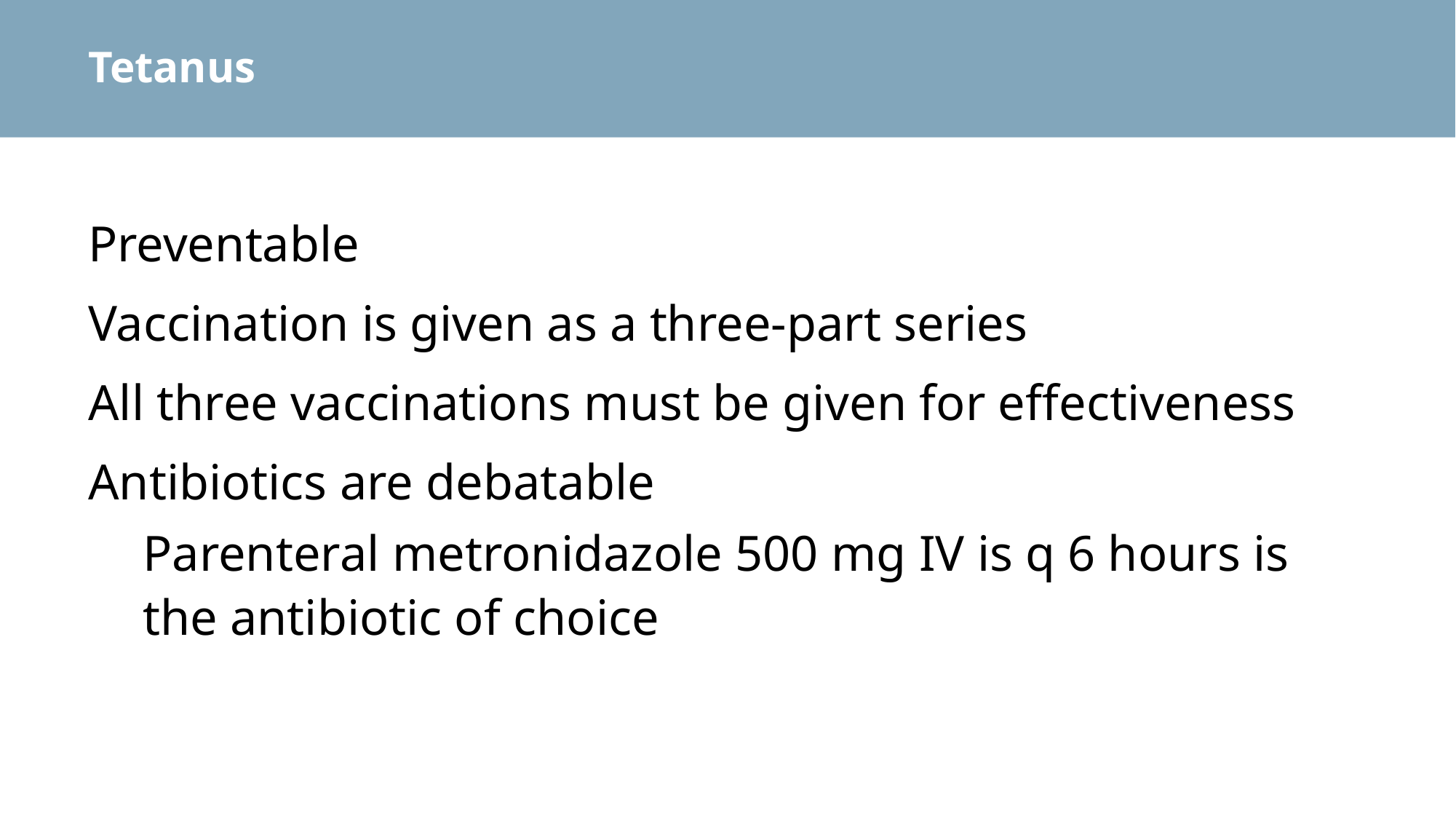

# Tetanus
Preventable
Vaccination is given as a three-part series
All three vaccinations must be given for effectiveness
Antibiotics are debatable
Parenteral metronidazole 500 mg IV is q 6 hours is the antibiotic of choice

## Slide 9
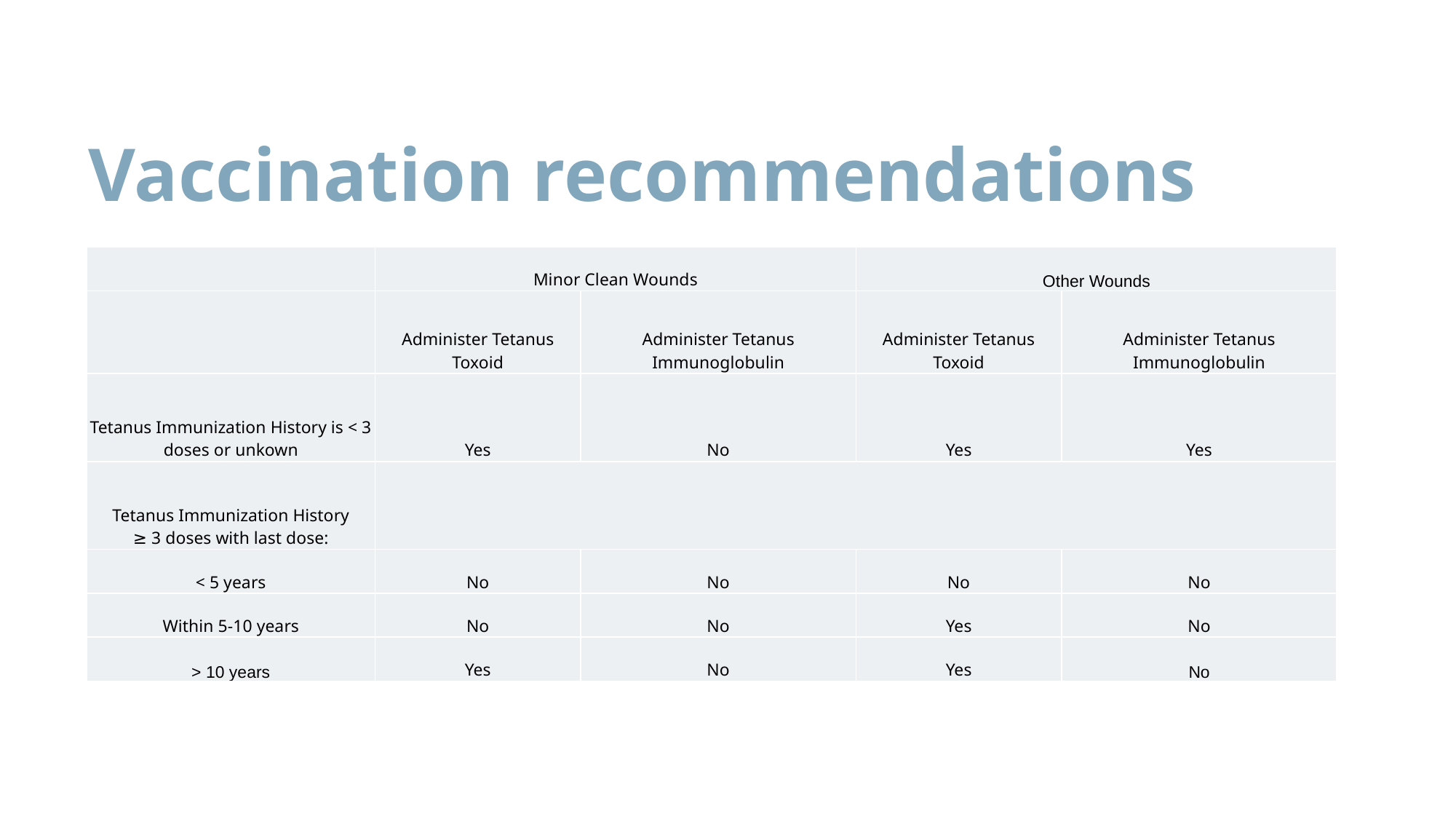

# Vaccination recommendations
| | Minor Clean Wounds | | Other Wounds | |
| --- | --- | --- | --- | --- |
| | Administer Tetanus Toxoid | Administer Tetanus Immunoglobulin | Administer Tetanus Toxoid | Administer Tetanus Immunoglobulin |
| Tetanus Immunization History is < 3 doses or unkown | Yes | No | Yes | Yes |
| Tetanus Immunization History ≥ 3 doses with last dose: | | | | |
| < 5 years | No | No | No | No |
| Within 5-10 years | No | No | Yes | No |
| > 10 years | Yes | No | Yes | No |

## Slide 10
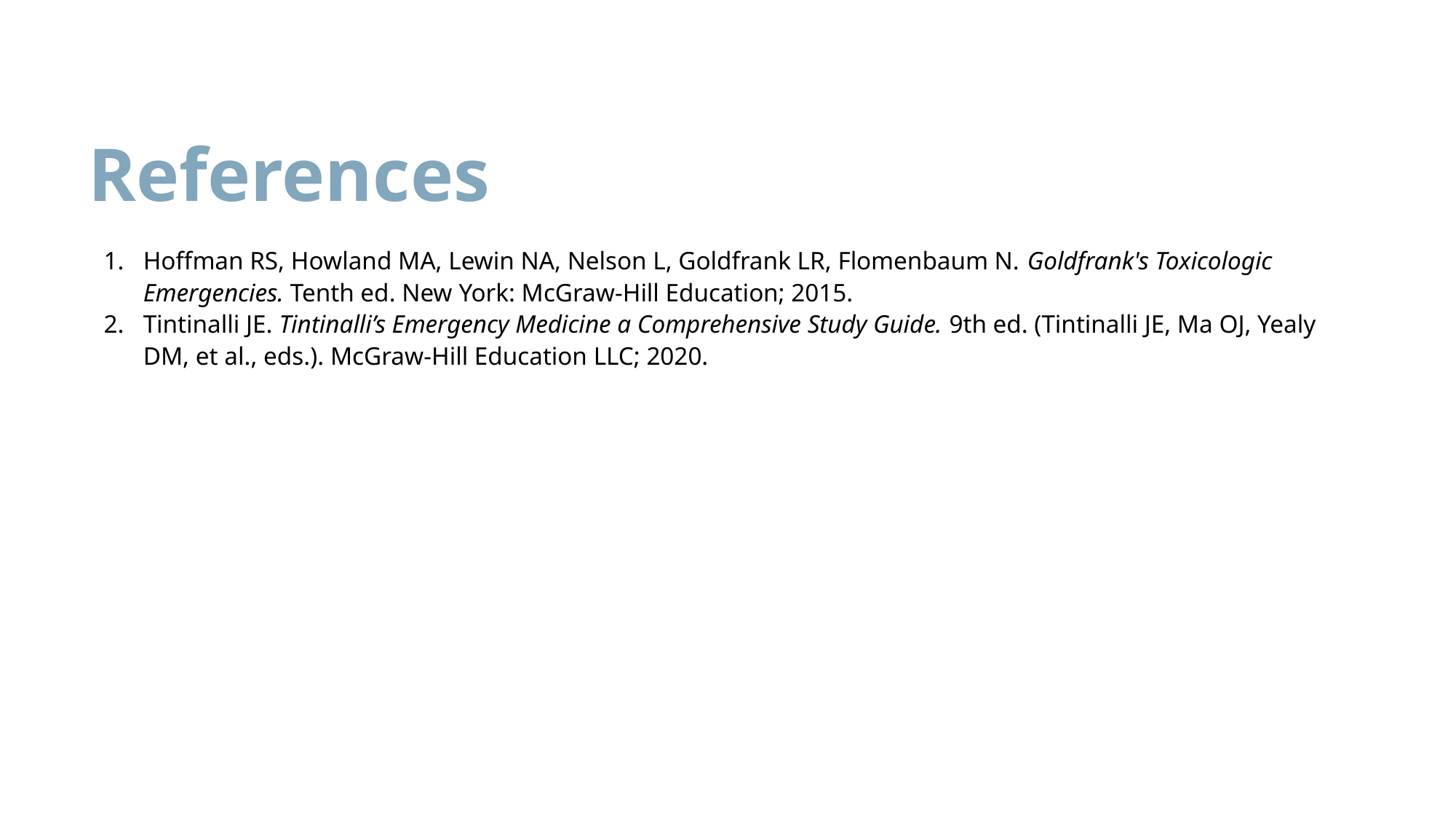

# References
Hoffman RS, Howland MA, Lewin NA, Nelson L, Goldfrank LR, Flomenbaum N. Goldfrank's Toxicologic Emergencies. Tenth ed. New York: McGraw-Hill Education; 2015.
Tintinalli JE. Tintinalli’s Emergency Medicine a Comprehensive Study Guide. 9th ed. (Tintinalli JE, Ma OJ, Yealy DM, et al., eds.). McGraw-Hill Education LLC; 2020.
