# Supplementary material for: Acute Muscle Rigidity Secondary to Tetanus: A Toxicology Simulation Case for Fourth-Year Medical Students
Source: MedEdPORTAL. 2024 Mar 29;20:11389. doi: 10.15766/mep_2374-8265.11389 (PMC10978813; doi:10.15766/mep_2374-8265.11389)
Supplement: Supplementary file 1 — Approach to Acid-Base Disturbances.pptxGlycine.pptxSimulation Images and Lab Values.docxSimulation Case.docxCritical Actions Checklist.docxDebriefing Materials.docxPre- and Posttest.docxSession Evaluation.docx [file mep_2374-8265.11389-s001.zip › C. Simulation Images and Lab Values.docx]

**Appendix C: Simulation Images and Lab Values**

All of the following images are author owned and created.

Instructions: Utilize these images as an aid for setting up the simulation. The lab values, EKG and chest Xray interpretation can be shown to learners when and if they ask for them during the course of the simulation.

**
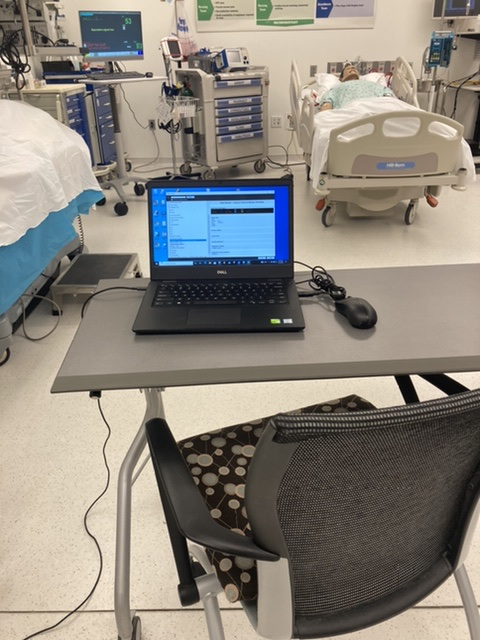
**

Control Panel

**
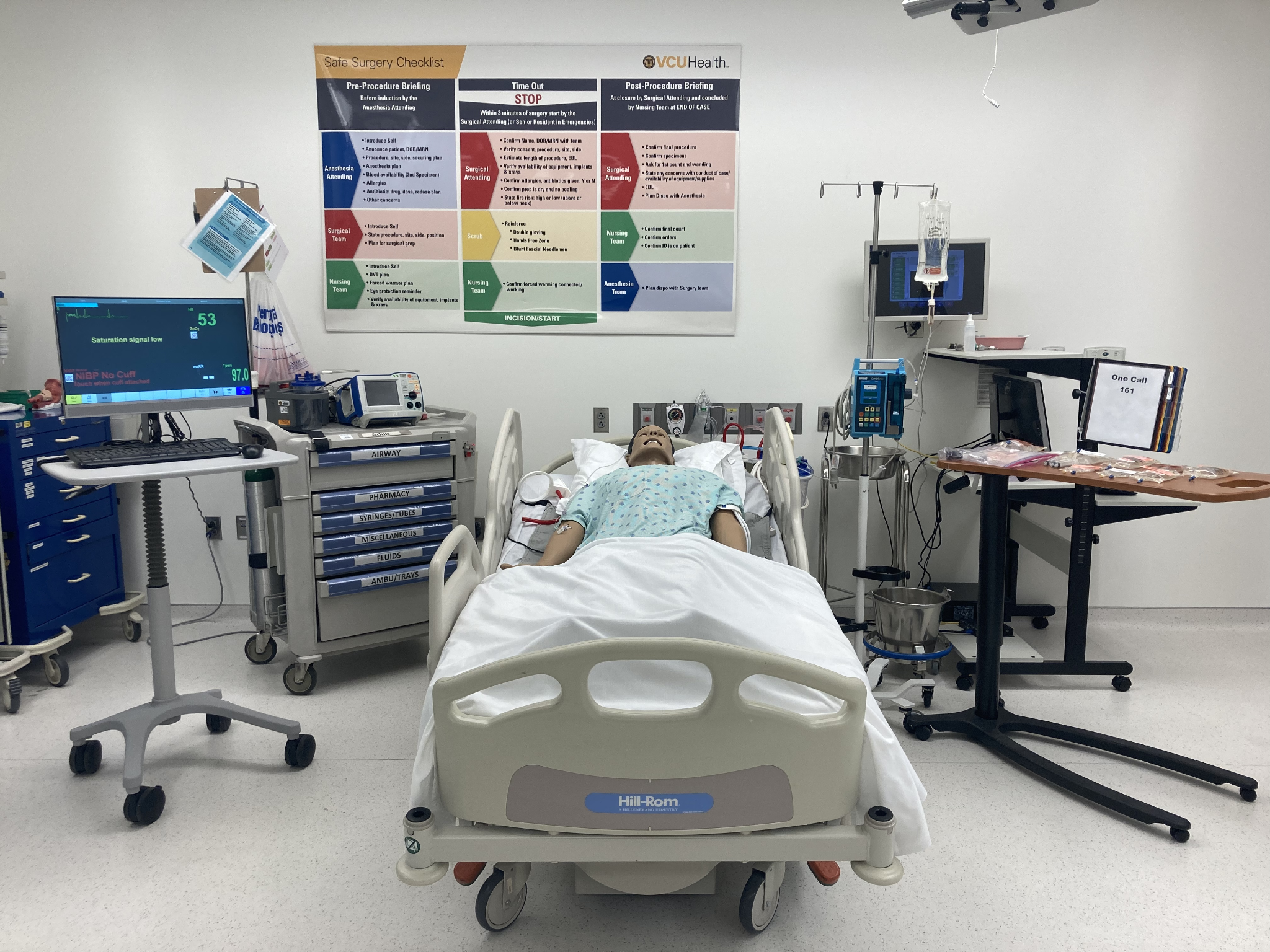
**

Mannequin and general setup


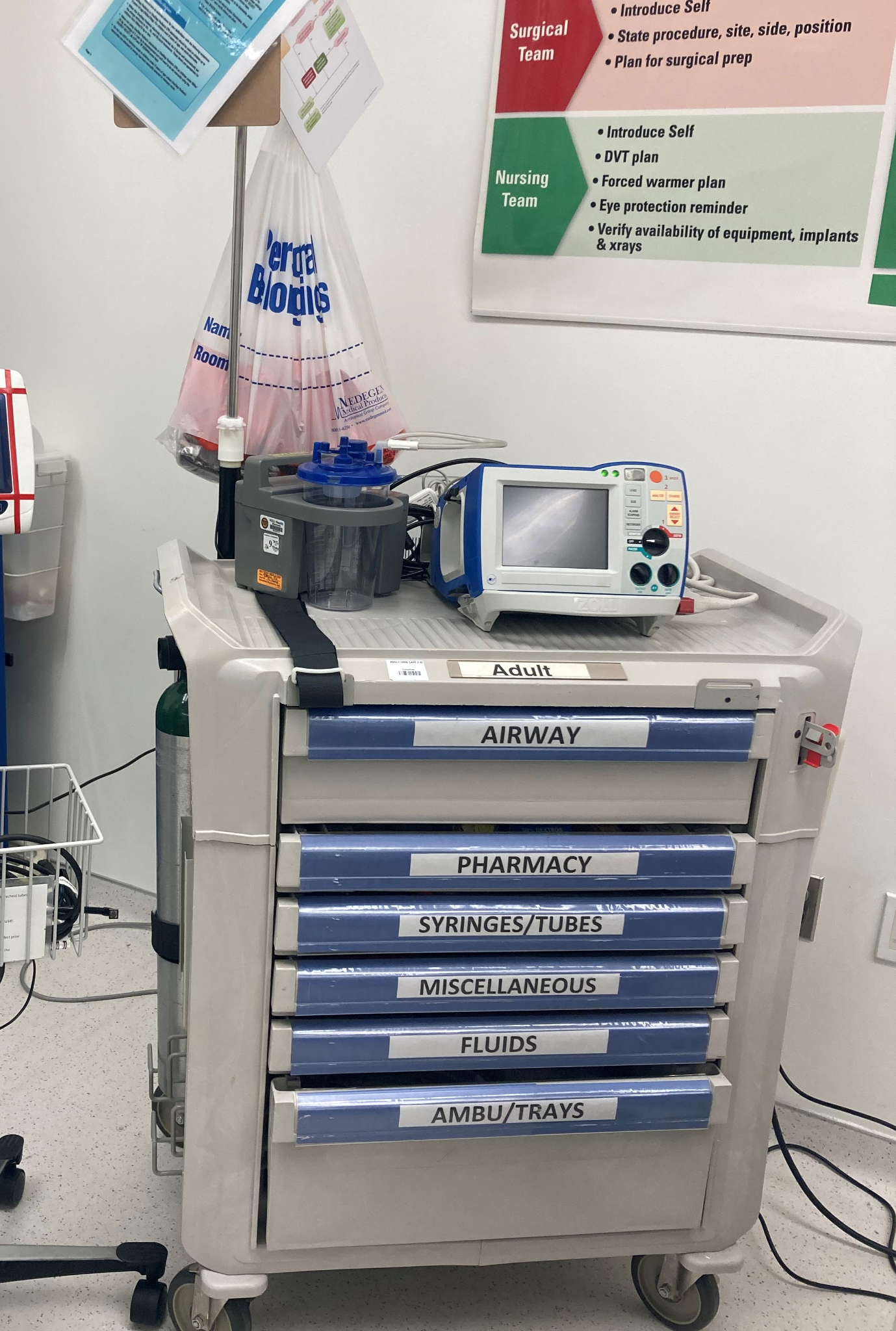


Airway cart with defibrillator


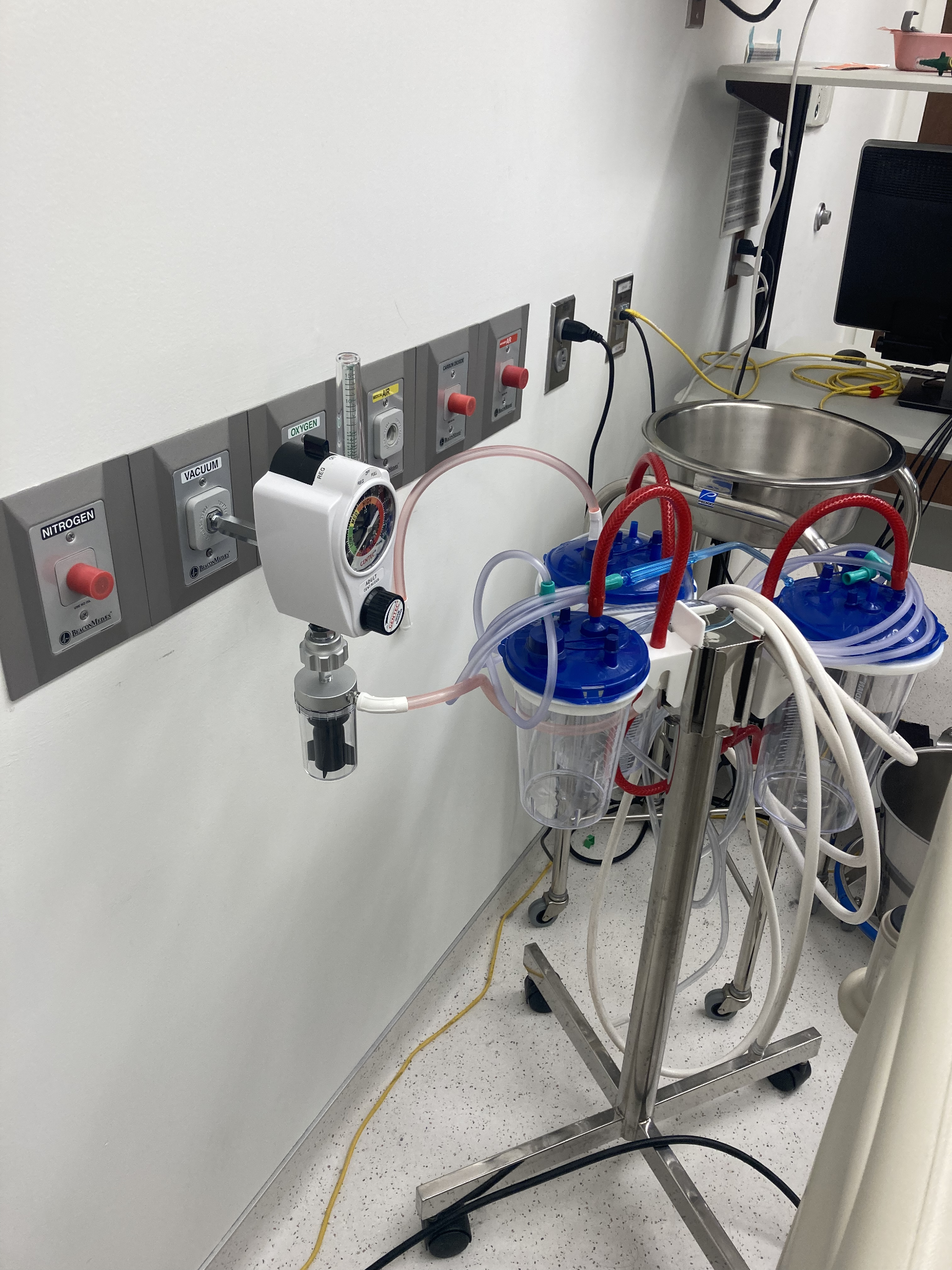


Wall with suction and oxygen


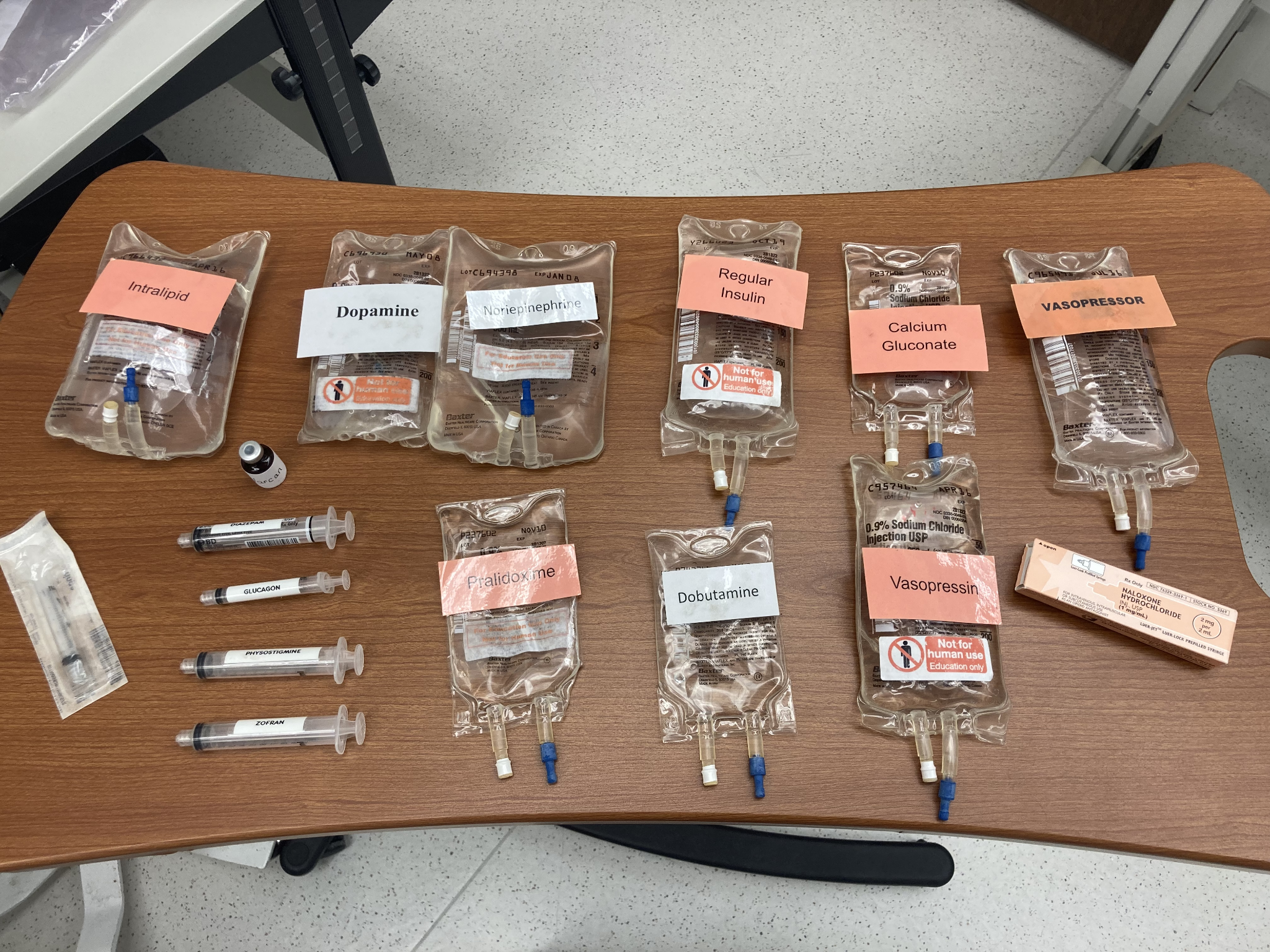


Pharmaceuticals station


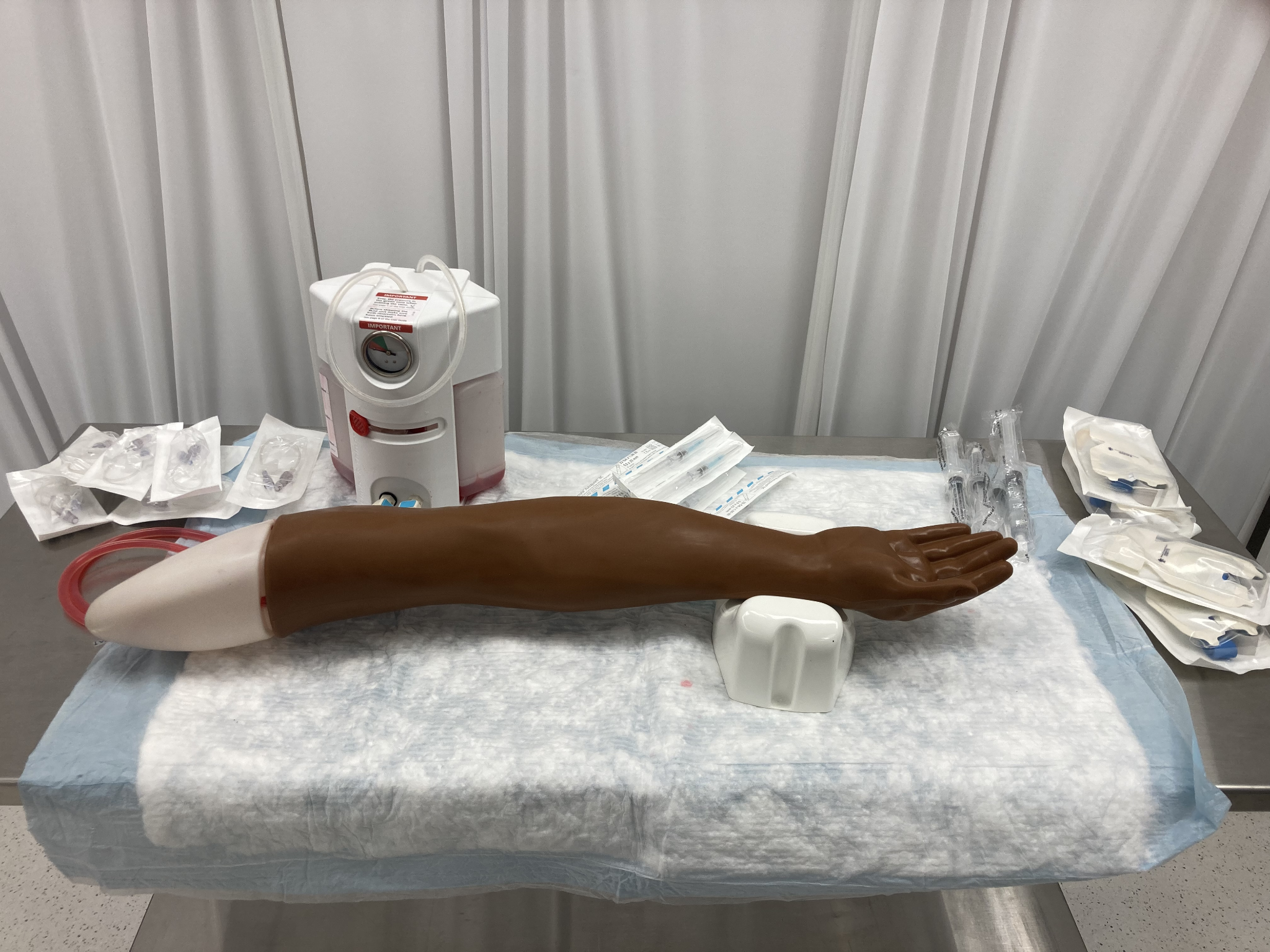


IV access station


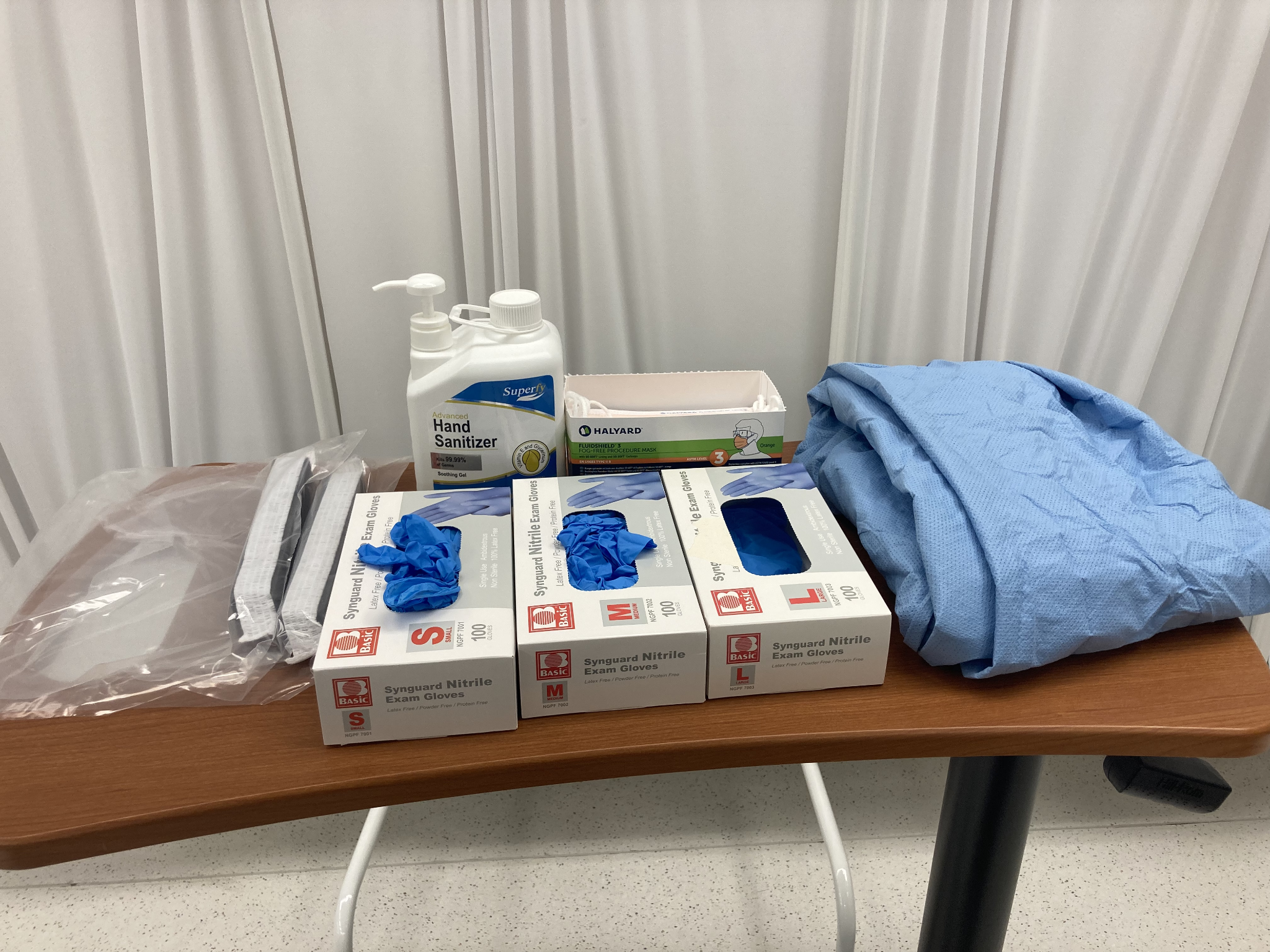


Personal protective equipment (PPE) station


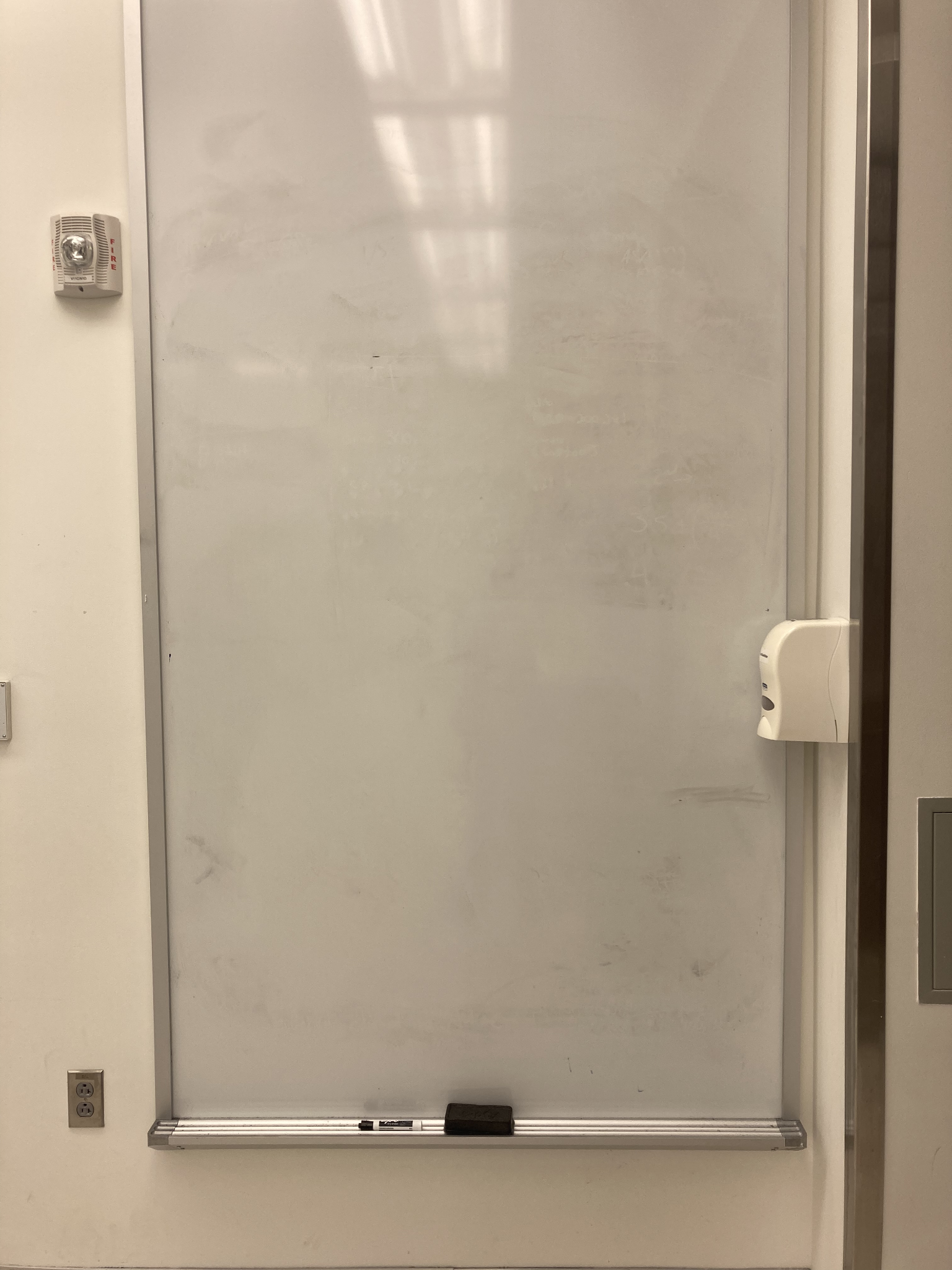


Documentation station

**LABORATORY VALUES**

**CBC**

Result Reference

WBC **19.8** 4.5-11 10e9/L

Hgb 12.3 12-16 g/dL

Hct 36.7 36-46%

Plt 218 150-450 10e9/L

**Basic metabolic Panel**

Result Reference

Sodium 143 136-145 mmol/L

Potassium **6.8** 3.5-5.1 mmol/L

Chloride 102 98-107 mmol/L

HCO_3_ **13** 22-30 mmol/L

BUN **77** 6-20 mg/dL

Creatinine **2.6** 0.8-1.2 mg/dL

Glucose **167** 65-99 mg/dL

Calcium **6.2** 8.0-10.6 mg/dL

**Hepatic panel**

Result Reference

Albumin 4.3 3.5-5.0 g/dL

T bili 0.9 0.0-1.0 mg/dL

D bili 0.2 0.0-0.2 mg/dL

Alk phos 66 35-109 U/L

AST **122**  0-37 U/L

ALT **156** 0-41 U/L

Total protein 6.7 6.4-8.3 g/dL

**Coagulation profile**

Result Reference

INR 1.0

PTT 26 22-33 sec

**Arterial Blood Gas (pre-intubation)**

Result Reference

pH **7.12** 7.31-7.41

pCO2 **76** 40-50 mmHg

P02 **33** 46-42 mmHg

HCO3 **13** mEq/L

BE **-10** -2 to +2

O2 saturation **54** 60-80%

Miscellaneous Labs

Result Reference

Magnesium 1.7 1.7-2.2 mg/dL

Phosphorus  **6.7** 2.5-4.5 mg/dL

Lactate **9.5** <2 mmol/L

CPK **61,398** 22-198 U/L

APAP – undetectable

ASA – undetectable

EKG – **Sinus tachycardia**, QRS 85, **QTc 575, peaked T waves**

CXR – (pre-intubation), **hypo-inflated lungs**, no acute pulmonary process
