# Supplementary material for: Acute Muscle Rigidity Secondary to Tetanus: A Toxicology Simulation Case for Fourth-Year Medical Students
Source: MedEdPORTAL. 2024 Mar 29;20:11389. doi: 10.15766/mep_2374-8265.11389 (PMC10978813; doi:10.15766/mep_2374-8265.11389)
Supplement: Supplementary file 1 — Approach to Acid-Base Disturbances.pptxGlycine.pptxSimulation Images and Lab Values.docxSimulation Case.docxCritical Actions Checklist.docxDebriefing Materials.docxPre- and Posttest.docxSession Evaluation.docx [file mep_2374-8265.11389-s001.zip › E. Critical Actions Checklist.docx]

**Appendix E: Critical Actions Checklist**

Instructions: Check items off the list if they are performed within 20 minutes of the simulation.

- Divide up roles amongst each other
- Place patient on cardiac monitor
- Make the decision to establish IV access
- Provide supplemental oxygen
- Obtain finger stick blood glucose
- Obtain EKG
- Start IV fluids
- Make the decision to perform endotracheal intubation
- Administer tetanus immunoglobulin for suspected tetanus
- Administer calcium gluconate for hyperkalemia
- Obtain chest X-ray
- Toxicology laboratory work
  - Acetaminophen level
  - Salicylate level
  - CBC
  - BMP, Mg, Phos
  - Hepatic panel
  - ABG
  - CPK
