# Supplementary material for: Acute Muscle Rigidity Secondary to Tetanus: A Toxicology Simulation Case for Fourth-Year Medical Students
Source: MedEdPORTAL. 2024 Mar 29;20:11389. doi: 10.15766/mep_2374-8265.11389 (PMC10978813; doi:10.15766/mep_2374-8265.11389)
Supplement: Supplementary file 1 — Approach to Acid-Base Disturbances.pptxGlycine.pptxSimulation Images and Lab Values.docxSimulation Case.docxCritical Actions Checklist.docxDebriefing Materials.docxPre- and Posttest.docxSession Evaluation.docx [file mep_2374-8265.11389-s001.zip › F. Debriefing Materials.docx]

**Appendix F: Debriefing Materials**

Instructions: Use this appendix to debrief the simulation with learners immediately following the simulation. We recommend spending at least 20 minutes on this debrief.

**Facilitator-led questions to ask learners at the start of the debrief following the conclusion of the simulation are as follows:**

1. How did the simulation feel?

2. What do you feel went well?

3. What would you do differently?

4. What are some things you noticed immediately about the patient?

5. Tell me about your thought process during the case. What did you think was happening to the patient?

6. What were some things you wanted to address about the patient early on? How did you manage the patient's ABCs (airway, breathing, circulation)?

7. Tell me about your choice of labs and medications that you ordered.

8. What was the patient's disposition? Why?

9. Please give a brief summary of the case overall, including first impressions, diagnosis, and interventions.

10. How would you apply what you've learned here in your future practice?

**This discussion may follow with a brief review of tetanus vaccine schedules, pathology, and management as follows:**

- Tetanus is caused by the exotoxin produced by the bacterium *Clostridium Tetani*, a gram-positive, anaerobic rod.
  - Left untreated, it is often fatal.
  - Tetanus prevents the release of glycine.
  - Glycine is an inhibitory neurotransmitter.
    - When it binds to its receptor, chloride shifts intracellularly. This causes hyperpolarization of the cell.
- Tetanus prevention
  - Tetanus is prevented by vaccination.
  - Vaccination is given as a three-part series. All three vaccinations must be given for effectiveness.
    - While this patient received the tetanus vaccination the previous week, he never received the original series.
  - If the patient has received the three-part vaccination series, but it has been between 5 and 10 years since receiving the last booster vaccine, he or she must receive a tetanus booster to prevent tetanus.
  - If the patient has not received the original vaccination series or only part of the original vaccination series, he or she must receive tetanus immunoglobulin as well as the first of the vaccination series followed by the second and third part of the vaccine series at 2 and 6 months respectively.
  - See table below for guidelines for the prevention of tetanus

|  | Minor Clean Wounds | | Other Wounds | |
| --- | --- | --- | --- | --- |
|  | Administer Tetanus Toxoid? | Administer Tetanus Immunoglobulin? | Administer Tetanus Toxoid? | Administer Tetanus Immunoglobulin? |
| Tetanus Immunization History is < 3 doses or unknown | Yes | No | Yes | Yes |
| Tetanus Immunization History  ≥ 3 doses with last dose: |  | | | |
| < 5 years | No | No | No | No |
| Within 5-10 years | No | No | Yes | No |
| > 10 years | Yes | No | Yes | No |

- Tetanus treatment
  - For the treatment of tetanus patients should receive the tetanus immunoglobulin as well as the first of the vaccination series followed by the second and third part of the vaccine series at 2 and 6 months respectively.
  - Antibiotics are debatable.
    - Parenteral metronidazole 500 mg IV every 6 hours is the antibiotic of choice.
- Lab derangements in rhabdomyolysis, and consequential EKG findings
  - Rhabdomyolysis causes elevated liver function tests as exemplified in this case.
  - Hypocalcemia
    - Secondary to calcium phosphate deposits on destroyed muscle cells in early rhabdomyolysis.
  - The QTc is prolonged because of hypocalcemia.
    - This causes phase 2 of repolarization to lengthen, as calcium channels will need to stay open longer. As phase 2 is part of repolarization, and QTc is associated with length of repolarization, it will be prolonged.
    - Tachycardia is protective from torsades de pointes due to forced speed of repolarization.
  - Hyperkalemia is secondary to rhabdomyolysis and renal failure.
    - With hyperkalemia, there is more potassium extracellularly. Normally, there is relatively more potassium intracellularly. This will result in a more neutralized potassium charge between the membranes.
      - For unclear reasons, this causes the potassium channels to become more sensitive, increasing the rate in which potassium leaves the cell. This manifests in peaked T waves.
    - First treat with calcium gluconate – 2,000 mg bolus should be given. This takes priority, as it will help stabilize the cardiac membranes.
    - Agents can be used to shift potassium intracellularly, including
      - Sodium bicarbonate; 1-2 amps may be considered
      - Insulin with dextrose
      - Beta-2 agonist (not recommended for this case – will worsen tachycardia)
    - Definitive removal of potassium from the body
      - Lasix (not recommended for this case – the patient needs aggressive IV fluids for rhabdomyolysis)
      - Dialysis
      - Lokelma
  - Rhabdomyolysis will result in an anion gap metabolic acidosis.
- Interpretation of the ABG is below:
  - pH is acidotic
  - HCO_3_ < 22 and _p_CO_2_ > 45; Primary process is both metabolic and respiratory acidosis
  - Use the following formulate to calculate the anion gap (AG):
    - AG = Na – (HCO_3_ + Cl)

AG = 143 – (13 + 102) = 28; elevated

- - Calculate delta-delta: [(Anion Gap) - 12] + HCO_3_
    - (28-12) + 13 = 29; Normal
  - Interpretation: Primary anion-gap metabolic acidosis and primary respiratory acidosis

**References**

1. Dzierba AL, Abraham P. A practical approach to understanding acid-base abnormalities in critical illness [published correction appears in J Pharm Pract. 2011 Oct;24(5):515]. *J Pharm Pract*. 2011;24(1):17-26. doi:10.1177/0897190010388153
2. Hoffman RS, Howland MA, Lewin NA, Nelson L, Goldfrank LR, Flomenbaum N. *Goldfrank's Toxicologic Emergencies.* Tenth ed. New York: McGraw-Hill Education; 2015.
3. Tintinalli JE. *Tintinalli’s Emergency Medicine a Comprehensive Study Guid*e. 9th ed. (Tintinalli JE, Ma OJ, Yealy DM, et al., eds.). McGraw-Hill Education LLC.,; 2020.
