# Supplementary material for: Acute Muscle Rigidity Secondary to Tetanus: A Toxicology Simulation Case for Fourth-Year Medical Students
Source: MedEdPORTAL. 2024 Mar 29;20:11389. doi: 10.15766/mep_2374-8265.11389 (PMC10978813; doi:10.15766/mep_2374-8265.11389)
Supplement: Supplementary file 1 — Approach to Acid-Base Disturbances.pptxGlycine.pptxSimulation Images and Lab Values.docxSimulation Case.docxCritical Actions Checklist.docxDebriefing Materials.docxPre- and Posttest.docxSession Evaluation.docx [file mep_2374-8265.11389-s001.zip › D. Simulation Case.docx]

| **Appendix D: *MedEdPORTAL* Simulation Case**  **SIMULATION CASE TITLE: Acute Muscle Rigidity Secondary to Tetanus: a Toxicology Simulation Case for Fourth-Year Medical Students**  **AUTHORS: Elizabeth Mangin, BA and Michelle Troendle, MD, FACEP**  **LEARNER AUDIENCE: Fourth-Year Medical Students** | |
| --- | --- |
| **PATIENT NAME: Gabriel Garcia**  **PATIENT AGE: 27**  **CHIEF COMPLAINT: “My husband can’t breathe” per spouse**  **PHYSICAL SETTING: Simulation Lab of Emergency Department** | |
| Instructions: This case template is to be used as a step-by-step guide on how to create the simulation. Learners have 20 minutes to work through the simulation and complete all the items on the critical actions list. | |
| **Brief narrative description of case** | A 27-year-old male patient without previous tetanus vaccinations presents to the emergency department with a chief complaint of muscle rigidity 1 week after sustaining a bite by a stray dog. The learner goals are to initiate the appropriate workup and management of a patient with acute muscle rigidity and to recognize the risk factors for tetanus, the appropriate administration of tetanus vaccine and immunoglobulin, and the associated sequela of respiratory acidosis from restricted chest wall movement with the need for endotracheal intubation for respiratory compromise. Secondary goals are to understand the medical sequela of rhabdomyolysis from muscle rigidity secondary to tetanus. |
| **Primary Learning Objectives** | - Describe the pathophysiology of tetanus - Recognize associated physical exam findings of tetanus - Discuss the appropriate algorithm of administration of tetanus vaccine and immunoglobulin for the prevention of tetanus - Discuss the appropriate algorithm of administration of tetanus vaccine and immunoglobulin for the treatment of tetanus - Develop a differential diagnosis for acute muscle rigidity - Identify respiratory compromise secondary to rigid chest wall requiring endotracheal intubation - Identify rhabdomyolysis secondary to muscle rigidity |
| **Critical Actions** | 1. Place patient on cardiac monitor 2. Make the decision to establish IV access 3. Provide supplemental oxygen 4. Obtain finger stick blood glucose 5. Obtain EKG 6. Start IV fluids 7. Make the decision to perform endotracheal intubation 8. Administer tetanus immunoglobulin for suspected tetanus 9. Administer calcium gluconate for hyperkalemia 10. Obtain chest X-ray 11. Toxicology laboratory work     1. Acetaminophen level     2. Salicylate level     3. CBC     4. BMP, Mg, Phos     5. Hepatic panel     6. ABG     7. CPK |
| **Learner Preparation or Prework** | Learners should be given the lectures “Approach to Acid-Base Disturbances” (Appendix A) and “Glycine” (Appendix B) for review up to a week before the simulation. Before the start of the case, they should take the pretest (Appendix G). Ideally students should know how to place an IV and intubate before the case or receive information on this prior to the start of the simulation. However, performing these procedures successfully is not a critical action. |

| **Initial Presentation** | | | |
| --- | --- | --- | --- |
| **Initial vital signs** | Temperature: (Oral) 39.1 C (102.4 F), Heart Rate 122 bpm, Respiration Rate 8 br/min, SpO2 93 %, Blood Pressure 163/101 mmHg | | |
| **Overall Setting and Appearance** | Patient is lying on a stretcher, conscious but distressed and in pain, in a simulated emergency department exam room. He appears to be struggling to breathe. | | |
| **Standardized Participants (and their roles in the room at case start**) | A standardized participant portraying the patient’s spouse at the bedside gives most of the history of present illness, extended history and review of systems.  The facilitator running the case sits at the control panel, alters the patient settings and voice accordingly. If the “spouse” is not present, the facilitator can also take on that role and give the history. | | |
| **HPI** | The following history is provided by the patient’s spouse and given without prompting:  “He started to complain that his mouth and hands were becoming stiff yesterday evening. Then he felt like the pain was going through his entire body. He feels stiff all over now. He was in the emergency department last week after he got bit by a dog. They rinsed out the bite and gave him three shots. One I think was a tetanus booster, and the other 2 were for rabies. They gave him an antibiotic which he’s been taking every day since then. They also told him to come back a few times this week for more rabies shots, which he did. I don’t know what’s happening to him. He seems like he’s in a lot of pain, and he just started having trouble breathing. I don't know what’s wrong with him. Does this have anything to do with the dog bite, or the medications they were giving him?”  Social history: Does not smoke, consume alcohol, or do recreational drugs  Born and raised in Mexico | | |
| **Past Medical/Surgical History** | **Medications** | **Allergies** | **Family History** |
| None | Augmentin 875/125 mg  No chronic medications | None | Unknown |
| **Physical Examination** | | | |
| **General** | Patient has opisthotonic posturing. He is in moderate distress and appears to be in pain | | |
| **HEENT** | Normocephalic, atraumatic, pupils 4 mm, equally round and reactive to light, extraocular movements with severe limitation in range in all directions, moderately dry mucous membranes, perioral cyanosis, trismus present | | |
| **Neck** | Rigid, trachea midline, carotid pulses 2+ bilaterally | | |
| **Lungs** | Bradypnea, shallow respirations, otherwise clear to auscultation bilaterally with equal breath sounds | | |
| **Cardiovascular** | Sinus tachycardia, no murmurs, rubs, or gallops, 2+ radial and dorsalis pedis pulses bilaterally | | |
| **Abdomen** | Rigid, diffusely tender to palpation, decreased bowel sounds | | |
| **Neurological** | Awake, alert, rigidity in all four extremities but able to move extremities with limited range of motion, Glasgow Coma Score 12 (eyes 4, verbal 2, motor 6 -limited verbal sound secondary to trismus) | | |
| **Skin** | Diaphoretic, warm, multiple well healing puncture wounds to posterior left calf, no surrounding erythema or purulence | | |
| **GU** | Normal male external genitalia | | |
| **Psychiatric** | Unable to assess | | |

| **Instructor Notes - Changes and CASE Branch Points** | | |
| --- | --- | --- |
| **Intervention / Time point** | **Change in Case** | **Additional Information** |
| Within 2 minutes  Participants place patient on 2-6 L/min O_2_ by nasal cannula or 10-15 L/min O_2_ by non-rebreather | Patient’s O_2_ saturation increases to 94-95%; however, the patient continues to be in respiratory distress |  |
| Within 2 minutes  Patient placed on cardiac monitor | If patient placed on monitor, vital signs will populate | If patient is not placed on monitor, examiner states that vital signs have not been obtained since arrival |
| Within 2 minutes  IV access procedure started or verbalize they would like IV placed | If students place IV, medications may now be administered | If students have not attempted IV procedure or have not stated they would like one placed, examiner asks how medications will be administered |
| Within 2 minutes  Students verbalize they would like finger stick blood glucose | If students request finger stick blood glucose, examiner informs students of result | If finger stick blood glucose not requested, examiner asks if there are any bedside tests they would like |
| Within 2 minutes  Students verbalize they would like to obtain EKG | If EKG requested, results show peaked T waves | If no EKG obtained, examiner asks what heart rhythm the patient has |
| 5 minutes into case  Students administer calcium gluconate for suspected hyperkalemia due to peaked T waves | If calcium gluconate administered, no change in EKG | If calcium gluconate not administered, the examiner alerts students to a now wide-complex tachycardia. If students do not administer calcium gluconate, patient goes into cardiac arrest |
| 5 minutes into the case | Respiratory status begins to decline (decreased respirations and O_2_ saturation) if the patient has not been intubated | Facilitator alerts the learners to the patient’s decreased respiratory rate and O_2_ saturation; if endotracheal intubation not performed, patient goes into cardiac arrest |
| 7 minutes into case  CXR obtained after endotracheal intubation | If obtained, endotracheal tube in proper position | If no CXR performed after intubation, oxygen saturation does not go above 90% and breath sounds are only heard only on the right. Examiner asks if the endotracheal tube has been confirmed to be in proper position. |
| 7 minutes into the case | Heart rate increases to 130s-140s if IV fluids have not been given | Facilitator alerts the learners to the patient’s tachycardia |
| 10 minutes into the case | Increasing difficulty ventilating the patient if tetanus immunoglobulin has not given | Facilitator alerts the learners to the difficult ventilation |
| No OG tube or foley placed by the time MICU called |  | Facilitator, acting as medical ICU admissions, asks if the patient has an OG tube or foley in place before admitting |

**Ideal Scenario Flow**

Before the case begins, a group of 3-5 students assign each other the following roles, which can be combined as necessary: case leader, airway management, IV access, scribe, pharmacy, and cardiac monitor placement. The learners enter the room to find a patient in distress with opisthotonic posturing on the stretcher. They immediately place the patient on a cardiac monitor and obtain a full set of vital signs followed by a finger-stick blood glucose, EKG, and supplemental oxygen via nasal cannula and/or non-rebreather. They then perform a history and physical exam, and note bradypnea, chest rigidity, and shallow respirations. On noting his respiratory distress, they obtain IV access and decide to intubate with a non-depolarizing agent. They confirm proper endotracheal tube placement, secure it in place, and obtain a chest Xray and post-intubation ABG. They also place an OG tube and a foley catheter. Due to his tachycardia, the learners then decide to start IV fluids. They order labs including a CBC, BMP, magnesium, phosphate, LFT’s, salicylate and acetaminophen level, and a CPK. Potassium returns elevated, and EKG shows peaked T waves, and so they decide to administer calcium gluconate and agents that might shift the potassium intracellularly such as insulin with dextrose or sodium bicarbonate. The labs then return with an elevated CPK, and they decide to initiate aggressive IV hydration and benzodiazepines for muscle relaxation. Due to the patient’s muscle contractions, hyperthermia, respiratory distress, rhabdomyolysis and metabolic derangements coupled with his unvaccinated status, learners come to the conclusion that he has tetanus. They administer tetanus immunoglobulin and the first of the tetanus vaccination series. They also correct electrolytes as needed, continuous IV fluids, continued sedation, and serial labs. They request the patient be admitted to the medical ICU.

**Anticipated Management Mistakes**

1. Failure to recognize the need for calcium gluconate following hyperkalemia: Some learners did not immediately realize the need for calcium gluconate as a cardioprotective agent to prevent hyperkalemia-induced arrhythmia.
2. Forgetting the need for an OG tube and foley following intubation: Many learners forgot that when intubating someone, they must also receive an OG tube and foley.
3. Failing to ask about the original tetanus vaccination series: Learners may assume this patient received the tetanus vaccine series because the majority of the population has received it; while vaccinations are frequently required to attend school in the United States, other countries may have a different set of regulations. The patient was born and raised in Mexico, and he did not receive his original series of tetanus vaccination.
