# Supplementary material for: Acute Muscle Rigidity Secondary to Tetanus: A Toxicology Simulation Case for Fourth-Year Medical Students
Source: MedEdPORTAL. 2024 Mar 29;20:11389. doi: 10.15766/mep_2374-8265.11389 (PMC10978813; doi:10.15766/mep_2374-8265.11389)
Supplement: Supplementary file 1 — Approach to Acid-Base Disturbances.pptxGlycine.pptxSimulation Images and Lab Values.docxSimulation Case.docxCritical Actions Checklist.docxDebriefing Materials.docxPre- and Posttest.docxSession Evaluation.docx [file mep_2374-8265.11389-s001.zip › G. Pre- and Posttest.docx]

**Appendix G: Pretest and Posttest**

Instructions: Please allow learners 10 minutes to take this test before the simulation, and 10 minutes after the conclusion of this simulation. It is the same test to be taken before and after the simulation.

1. Binding of Glycine to its receptor causes:
   1. **Influx of Cl, causing hyperpolarization**
   2. Efflux of Cl, causing depolarization
   3. Efflux of K, causing hyperpolarization
   4. Influx of K, causing depolarization

Glycine causes Cl to influx from the postsynaptic neuron, leading to hyperpolarization.

1. Glycine is a cofactor for which neurotransmitter?
   1. Adenosine
   2. GABA
   3. Norepinephrine
   4. **Glutamate**

For successful agonism of glutamate, glycine is required as a cofactor on the postsynaptic glutamate receptor.

1. Tetanus:
   1. **Prevents the release of glycine from the presynaptic neuron**
   2. Prevents the binding of glycine on the postsynaptic neuron
   3. Inhibits the reuptake of glycine
   4. Prevents the synthesis of glycine

Tetanus prevents the release of glycine from the presynaptic neuron.

1. The physical exam finding caused by a grinning secondary to abnormal facial spasms is called:
   1. Opisthotonus
   2. Clonus
   3. **Risus sardonicus**
   4. Chvostek sign

This physical exam finding is called risus sardonicus, which is abnormal, prolonged spasm of the facial muscles that appears to produce grinning.

1. The physical exam caused by severe hyperextension of the head, neck and back is called:
   1. Clonus
   2. **Opisthotonus**
   3. Risus sardonicus
   4. Chvostek sign

This is opisthotonus, which is hyperextension of the head, neck and back.

1. Tetanus may result in:
   1. Hypocapnia
   2. **Hypercapnia**
   3. No effect on the carbon dioxide level
   4. None of the above

Tetanus will result in hypercapnia secondary to inability of the thorax to expand.

1. The mechanism of action of strychnine is:
   1. **Antagonizes glycine on the postsynaptic neuron**
   2. Prevents the release of glycine from the presynaptic neuron
   3. Inhibits the reuptake of glycine in the presynaptic neuron
   4. Inhibits the breakdown of glycine

Strychnine is an antagonist of glycine on the postsynaptic receptor.

1. Strychnine poisoning may result in:
   1. Rhabdomyolysis
   2. Respiratory acidosis
   3. Opisthotonus
   4. **All of the above**

In strychnine poisoning, glycine is antagonized, which does not allow muscles to relax. The thorax will not be able to expand properly, causing respiratory acidosis. Opisthotonus, which is hyperextension of the neck and back, may be seen in strychnine poisoning. Another physical exam finding in strychnine poisoning is risus sardonicus, or prolonged, abnormal facial muscle contractions. Rhabdomyolysis occurs due to intense, prolonged contraction of muscles, resulting in muscle breakdown.

1. An asymptomatic patient without prior vaccination stepped on a rusty nail.  You should:
   1. Administer tetanus immunoglobulin IM
   2. Administer first of three tetanus toxoid vaccine IM
   3. Administer metronidazole 500 mg IV
   4. **Both A & B**

When a patient without previous vaccination has an exposure to tetanus, the patient must receive both the tetanus immunoglobulin and the first of the three tetanus toxoid vaccines in the series IM at different sites (such as right or left arm).  The patient will still need to receive the second and third series of the vaccine at a later date for full immunization.  Antibiotics are debated and not routinely recommended.

1. An asymptomatic patient with prior tetanus vaccination steps on a rusty nail.  You should:
   1. Administer tetanus immunoglobulin
   2. Administer tetanus toxoid
   3. Administer metronidazole 500 mg IV
   4. **Determine when the last immunization was**
