# Supplementary material for: Acute Muscle Rigidity Secondary to Tetanus: A Toxicology Simulation Case for Fourth-Year Medical Students
Source: MedEdPORTAL. 2024 Mar 29;20:11389. doi: 10.15766/mep_2374-8265.11389 (PMC10978813; doi:10.15766/mep_2374-8265.11389)
Supplement: Supplementary file 1 — Approach to Acid-Base Disturbances.pptxGlycine.pptxSimulation Images and Lab Values.docxSimulation Case.docxCritical Actions Checklist.docxDebriefing Materials.docxPre- and Posttest.docxSession Evaluation.docx [file mep_2374-8265.11389-s001.zip › _Educational Summary Report_11389.pdf]

MedEdPORTAL® | The AAMC Journal of Teaching and Learning Resources

Original Publication 	Open Access

Acute Muscle Rigidity Secondary to Tetanus: A Toxicology Simulation Case for Fourth-Year Medical Students

Elizabeth Mangin*, Michelle Troendle, MD

*Corresponding author: elmangin@gmail.com

Abstract

Introduction: Tetanus is uncommon in the United States secondary to vaccination. However, vaccination hesitancy is increasing. This case challenges medical students to consider tetanus in the differential and understand its complications. **Methods:** Fourth-year medical students took a pretest on the neurotransmitter glycine and associated disease states. They received two 10-minute lectures on glycine and acid-base abnormalities. Students then participated in a simulation featuring a 27-year-old man bitten by a dog, resulting in tetanus. Required equipment included a mannequin with monitor, a defibrillator, and personal protective equipment. Critical actions consisted of learners dividing up roles amongst each other, using closed-loop communication, placing the patient on a cardiac monitor, choosing to establish IV access and intubate the patient, starting IV fluids, and administering tetanus immunoglobulin. The case ended after 20 minutes. Outcome measurements encompassed performance on a posttest and critical actions. **Results:** Twenty students participated. Mean pretest and posttest scores were 69.5 and 92.5, respectively ($p < .001$). All groups completed the items on the critical actions checklist within a 20-minute time frame. **Discussion:** Rising vaccine hesitancy may increase the likelihood of physicians encountering new cases of tetanus and require them to perform lifesaving management of a patient presenting with muscle rigidity. This simulation provides learners with hands-on experience caring for a patient with tetanus and muscle rigidity. It can improve their knowledge of recognition, assessment, and decision-making toward lifesaving management of tetanus by allowing them to practice their skills in a safe environment.

Keywords
Tetanus, Toxicology, Vaccination, Case-Based Learning, Emergency Medicine, Medical Toxicology, Pharmacology & Toxicology, Simulation

Educational Objectives

By the end of this activity, learners will be able to:

1. Describe the pathophysiology of tetanus.
2. Recognize associated physical exam findings of tetanus.
3. Discuss the appropriate algorithm of administration of tetanus vaccine and immunoglobulin for the prevention of tetanus.
4. Discuss the appropriate algorithm of administration of tetanus vaccine and immunoglobulin for the treatment of tetanus.
5. Develop a differential diagnosis for acute muscle rigidity.
6. Identify respiratory compromise secondary to rigid chest wall requiring endotracheal intubation.
7. Identify rhabdomyolysis secondary to muscle rigidity.

Citation:
Mangin E, Troendle M. Acute muscle rigidity secondary to tetanus: a toxicology simulation case for fourth-year medical students. *MedEdPORTAL*. 2024;20:11389.
https://doi.org/10.15766/mep_2374-8265.11389

Introduction

Clostridium tetani is a ubiquitous, gram-positive, anaerobic bacterium that exists in a sporulated form, most commonly in the soil.[1] When *C. tetani* infects an open wound, it releases a neurotoxic agent that blocks glycine neurotransmitter release at the inhibitory interneurons of the spinal cord, leading to diffuse muscle spasms and rigidity.[2] Tetanus is uncommon in the United States, with most cases occurring in unvaccinated or partially vaccinated patients.[3] The tetanus vaccination series is recommended for infants at 2, 4, and 6 months of age, with a booster dose at 15-18 months of age and at 4-6 years of age.[4] About 80% of children in the United States have received four or more components of the vaccine series.[5] The past several years have demonstrated an increase in vaccine hesitancy in the United States and throughout the world, and rates of childhood vaccination continued to decline during the COVID pandemic.[6,7]

Copyright © 2024 Mangin and Troendle This is an open-access publication distributed under the terms of the Creative Commons Attribution license.

Although the incidence of tetanus has remained consistent in recent years, vaccination rates against tetanus have definitively declined since the pandemic.[8,9] Tetanus remains rare, but given declining vaccination rates, medical professionals should be prepared for the possibility of encountering it and keep it in the differential. Encountering this low-frequency, high-acuity scenario can influence the ability of providers to recognize, diagnose, and treat tetanus in such a timely manner as is necessary to avoid bad outcomes.

There is a wide differential for the patient presenting with acute-onset muscle spasms and rigidity, including neuroleptic malignant syndrome, serotonin syndrome, autoimmune or infectious encephalitis, postictal state, and toxic or infectious causes.[10-12] Tetanus may not be considered due to assumption of its prevention with vaccination. In addition to rigidity, tetanus can cause autonomic disturbances, including tachycardia, hypertension, vasoconstriction, and diaphoresis.[13] Autonomic instability is not unique to tetanus, but may also be seen in such conditions as delirium tremens, neuroleptic malignant syndrome, serotonin syndrome, Guillain-Barre, and other causes, making differential diagnosis a challenge.[14-16] Muscle spasms, such as pharyngeal and laryngeal spasm, can lead to aspiration and airway obstruction.[17] Muscle rigidity can cause poor chest wall compliance and oxygenation, hypercapnia, hypoxia, and respiratory acidosis, requiring invasive airway management, use of paralytics, and ventilator support. Autonomic disturbances also occur, which require the use of benzodiazepines.[18] Diffuse spasms may lead to rhabdomyolysis, with the subsequent development of elevated lactate, anion-gap metabolic acidosis, hyperkalemia, hypocalcemia, and acute kidney injury, requiring electrolyte correction and administration of intravenous fluids.[17,19] Tetanus-specific management also includes wound care and administration of tetanus immunoglobulin.[20] It is critical for students, as future health care providers, to keep a broad differential diagnosis of muscle rigidity and autonomic instability, recognize tetanus as a potential cause, and address the life-threatening complications as well as medical sequelae of tetanus.

Health care professionals increasingly face the challenges of undervaccination. Our case prepares learners to gather a thorough history, particularly identifying vaccination gaps. They learn to consider the causes of acute muscle spasms and their medical sequelae and to initiate the distinct workup and stabilization of a patient with acute muscle spasms. There are limited learning cases on tetanus and its consequences if left unrecognized and untreated. To our knowledge, there are no similar cases currently published in *MedEdPORTAL*.

Given increasing vaccine hesitancy and the high-acuity, low-frequency nature of tetanus, a tetanus simulation is an asset to any toxicology curriculum.

Tetanus presentation and management are especially relevant for learners with an interest in pediatrics, emergency medicine, or internal medicine, as there is the possibility for them to encounter a similar case during their medical careers. This simulation allows learners to have hands-on experience with recognition, assessment, diagnosis, and management of a high-acuity, low-frequency, immersive tetanus scenario. As tetanus is a fulminant, fatal disease, these skills can be lifesaving and applicable not only to this case but also to other similarly presenting conditions. Furthermore, while this is a tetanus case, it covers topics on acid-base status, electrolyte abnormalities, rhabdomyolysis, and basic airway management that are essential to many fields of medicine.

Methods

Development

We implemented this case for fourth-year medical students during the last week of an optional 4-week elective titled Critical Care Toxicology. These students chose to take this elective to learn about toxicology and volunteered to participate in this nongraded simulation. A board-certified toxicologist created and ran the simulation. The methodology reflected the need to administer proper tetanus booster, vaccine series, and immunoglobulin administration. Therefore, the created case was a patient who had sustained a dog bite and received a booster for tetanus without having been asked about the initial tetanus vaccination series, rendering the booster ineffective. This allowed for reinforcement of learning via the administration of the tetanus vaccine series and immunoglobulin, as well as discussion why the tetanus booster failed. Prior to the simulation, learners watched the prerecorded Approach to Acid-Base Disturbances lecture (Appendix A) to review interpretation of arterial blood gas, as well as the prerecorded Glycine lecture (Appendix B), which highlighted tetanus and strychnine poisoning. Students also received instruction on how to perform intravenous cannulation and endotracheal intubation prior to the start of the simulation.

Institutional review board approval at Virginia Commonwealth University Health was waived due to the following: We initially developed this project as a method of quality improvement and not research, there was low risk of harm to all individuals who participated, participation was voluntary, and data were deidentified. We made learners aware before the simulation that participation and performance in the simulation or pretest and posttest would not affect their grade in the elective.

Copyright © 2024 Mangin and Troendle This is an open-access publication distributed under the terms of the Creative Commons Attribution license.

Equipment/Environment

We utilized a simulation lab room sectioned into the following stations: control panel, high-fidelity mannequin near the wall with suction and oxygen, airway cart with defibrillator and cardiac monitor, IV pole, pharmaceuticals, IV access, personal protective equipment, and documentation. We instructed students to bring their own stethoscopes.

The control panel contained a laptop that altered mannequin settings, a table, and a chair. We made the mannequin cyanotic in appearance with restricted chest rise and used voice controls to make the mannequin grimace in pain. The students were able to see a cardiac monitor controlled by the control panel, and the mannequin settings could be adjusted as needed.

Supplies located near the mannequin included the following:

- Suction setup with Yankauer and suction canister
- Oxygen setup with tubing and connector
- Airway cart containing nasal cannulas, capnography, non-rebreather masks, variously sized endotracheal tubes, variously sized Mac and Miller blades, glidescope, ambu-bag, 10-cc syringe, and bougie for anticipated difficult intubation
- Defibrillator with pads and monitor
- IV pole with infusion pump and primary IV tubing
- Cardiac monitor

Pharmaceuticals included the following:

- Vasopressors, including dopamine, norepinephrine, dobutamine, and vasopressin
- Antidotes, including calcium gluconate, glucagon, physostigmine, pralidoxime, and Narcan
- Regular insulin
- Diazepam
- Zofran
- Intralipid

The IV access station contained the following:

- IV arm part-task trainer on absorbent pad
- IV start kit
- 10-cc flush
- Needleless IV connector
- 22 gauge IVs (size recommended for task trainers)

Ultrasound could be used for IV access assistance at the examiner's discretion. However, if ultrasound guidance was used, angiocath needles were provided.

The personal protective equipment station contained the following:

- Gloves
- Face shields
- Face masks
- Hand sanitizer
- Sterile gowns

The documentation station contained a whiteboard with dry-erase markers and an eraser. Simulation pictures, lab values, and EKG results are shown in Appendix C.

Personnel

We informed participants that the patient's spouse was available to give a history. The facilitator running the case acted as the patient's spouse to give this history. The facilitator also sat at the control panel and altered the mannequin settings accordingly.

Implementation

Before the simulation, learners watched prerecorded lectures on acid and base disturbances (Appendix A) and glycine (Appendix B). They were able to access these lectures 1 week before the simulation to allow time for independent review. We presented the case to learners in simulation format (Appendix D) with accompanying simulation images (Appendix C). The critical actions checklist (Appendix E) assessed whether learners fulfilled all necessary interventions. We programmed the simulation software per the case file (Appendix D) and prepared the room with the items described in the Equipment/Environment section above prior to testing. The facilitator controlled the simulation software as described in Appendix D. The facilitator projected lab values and EKG interpretation (Appendix C) requested by learners onto a screen via PowerPoint. Students completed an 8-minute debrief following the simulation (Appendix F). Before and after the simulation, learners took an online pretest and posttest (Appendix G) to assess their knowledge of tetanus and glycine. The pretest and posttest were devised by the same board-certified toxicologist who created this case.

We divided learners into teams of three to five students. The learners entered the simulation room and delegated the following roles: case leader, airway management, IV access, scribe, pharmacy, and cardiac monitor placement. The facilitator informed learners about the patient's presentation, and learners could ask questions throughout the duration of the simulation. On encountering the patient, learners began their assessment by actions such as placing him on a cardiac monitor and obtaining vitals, a finger-stick blood glucose (FSBG), and an EKG. Given his

Copyright © 2024 Mangin and Troendle This is an open-access publication distributed under the terms of the Creative Commons Attribution license.

respiratory distress, the students chose to intubate after obtaining a brief history and physical. They started a benzodiazepine drip to treat his muscle spasms and ordered labs of their choosing. They administered tetanus immunoglobulin and the first tetanus vaccine due to concern for tetanus. At the conclusion of the case, they called to admit the patient to the medical intensive care unit. We expected teams to perform all actions in the critical actions checklist within 20 minutes. The facilitator observed the teams during the course of the simulation for completion of the items on the critical actions checklist. Debriefing commenced at the end of the simulation.

Debriefing

The debriefing session lasted 8 minutes. The facilitator led a discussion of the simulation using the educational objectives as a framework. The facilitator first asked the learners what they felt had gone well and what they wished they could have improved.

Examples of questions to facilitate a general overview of the case were as follows:

- How did the simulation feel?
- How would you apply what you have learned here in your future practice?

The facilitator then highlighted points in the critical actions checklist and why the actions were necessary. During this time, the facilitator utilized the debriefing sheet (Appendix F) to discuss important points about the pathophysiology and treatment of tetanus.

Assessment

We assessed learners using two methods. First, students took a multiple-choice pretest (Appendix G) to determine their baseline knowledge of glycine and its associated disorders. After the lectures and subsequent simulation, students took a posttest (Appendix G), which had the same content as the pretest. We based test questions primarily on the physiology of glycine, pathophysiology and physical exam findings of tetanus, and abnormal lab work associated with tetanus. We compared means of the pretest and posttest scores with a paired t-test. Assessment also occurred during the simulation based on performance of critical actions. We developed the checklist using necessary, lifesaving actions for a patient presenting with tetanus.

Results

Between July 2020 and November 2022, 20 fourth-year medical students participated in the lecture, simulation, and evaluation. All reported that they had listened to the required lectures before the simulation and that the lectures were helpful in preparing for the case. Every student who participated in the simulation completed the pretest and posttest, for a 100% participation rate. Results are summarized in the Table. Mean pretest score was 69.5 ($SD = 18.5$), and mean posttest score was 92.5 ($SD = 9.7$). A paired two-tailed t test was applied, with $p < .001$ (95% confidence interval: -0.3133 to -0.1467).

We ran this simulation five times. All teams completed the items on the critical actions checklist within 20 minutes. We gathered feedback from students during the postsimulation debrief, and the comments that follow were summarized by the facilitator after the debrief. They are not verbatim. Several learners commented that they enjoyed the holistic aspects of the case. They appreciated that the case highlighted the maintenance of airway, breathing, and circulation first and that they could practice various hands-on skills such as intubation and IV line access, which would be necessary during residency. They also enjoyed the review of tetanus vaccination and the novelty of a tetanus-based case. Students felt the case was advanced and could be suited for residency-level training. However, they enjoyed the challenge of the complex case and the myriad of responsibilities they were given during the course of this simulation.

Discussion

This case was developed for a 4-week fourth-year medical student toxicology elective. We constructed the case to address gaps in medical knowledge that might occur due to lack of opportunity to care for patients with tetanus clinically. We implemented the case during the last week of the elective

Table. Pre- and Posttest Scores for Medical Students

Pretest	Posttest
80	100
90	90
40	90
60	70
80	100
80	90
30	100
70	100
90	100
70	90
80	90
60	100
60	100
80	100
80	90
50	90
40	70
80	100
100	100
70	80

Copyright © 2024 Mangin and Troendle This is an open-access publication distributed under the terms of the Creative Commons Attribution license.

course. Before implementation, we instructed students how to perform IV access and intubation in the simulation lab so that they would have the opportunity to practice them during the simulation case. However, knowledge of these skills was not necessary to complete the simulation, and we allowed students to simply verbalize the need for IV cannulation or intubation to fulfill the corresponding critical actions. Their evaluation was based on objective data as described above.

To our knowledge, there are no other cases available in *MedEdPORTAL* that focus on the review, diagnosis, and treatment of tetanus. While tetanus remains rare, rising vaccine hesitancy necessitates that health care providers keep it in the differential. As tetanus is associated with high mortality, it is important that students learn how to manage it. This case is challenging due to the multiple layers involved. Students must first demonstrate an understanding of the pathophysiology of tetanus and include it in their differential for muscle rigidity. Students must also perform the initial assessment of the patient by obtaining a focused history and physical exam while simultaneously initiating management with placement on a monitor, establishing IV access, obtaining an EKG and FSBG, and providing supplemental oxygen. Finally, students must recognize and treat the underlying disorder plus the myriad of consequences associated with muscle rigidity, including respiratory acidosis, rhabdomyolysis, lactic acidosis, anion-gap metabolic acidosis, renal failure, and hyperkalemia. This simulation case allows medical students to practice skills necessary for residency in a safe learning environment.

While we used this case during a toxicology elective, the course content is relevant to areas of internal medicine, pediatrics, and emergency medicine and can be considered during these rotations. For learners who do not wish to participate in the simulation, learning can still be achieved via viewing the lectures and taking the posttest. The case can be expanded or abridged as needed depending on learners' needs and goals. For example, one could abridge the case to highlight only the management of airway, breathing, and circulation in a patient with diffuse muscle spasms. The case can also be simplified depending on learners' needs. For example, rather than requiring learners to identify the diagnosis of tetanus, they can be prompted with the diagnosis at the start of the case. The facilitator can also prompt them should they need guidance at any point in the simulation. If this simulation is incorporated into a course for a grade, learners could receive partial credit for this simulation if they require prompting.

We kept group sizes small, limiting teams to three to five learners at a time. Advantages of smaller groups include focused attention from the facilitator, enhanced responsibility as members have to take on at least one essential role during the simulation, and easier communication amongst team members due to the smaller team size. A disadvantage is that fewer students are able to participate in the simulation at one time. Smaller groups are also more time-consuming on the part of the facilitator, since maintaining small team sizes requires them to facilitate more simulations overall. Additionally, learners may feel more individual pressure in smaller teams. We feel that smaller groups are ideal, with five being the maximum number of learners at a time. However, as long as each learner has a role, team sizes can be expanded or reduced depending on the needs of the learners and the resources of the simulation lab. For example, if increasing the size to six team members, teams could add the role of co-team lead or history-taker.

Expanded evaluation can occur by applying the approach and critical actions to a separate case. Furthermore, facilitators can distribute an anonymous survey to learners immediately following the simulation to evaluate satisfaction and suggested areas of improvement. A weakness of our case is that we failed to create a written session evaluation, but we have included an example of one that can be used in the future (Appendix H). Also in the future, we would employ a separate person to alter the mannequin settings rather than requiring the case facilitator to do so, so that the facilitator's full attention can be on the learners' actions during the case. Ideally, we would also utilize a separate standardized participant to act as the patient's spouse and give a history to the learners at bedside during the simulation. The debriefing session lasted only 8 minutes due to time constraints for the simulation. However, we also feel that 8 minutes did not sufficiently permit discussion of the intricacies of the case while allowing time for student feedback and questions. We recommend increasing the debrief time to at least 20 minutes so that the case can be adequately reviewed.

Simulated cases do have limitations. First, one must have access to a simulation center. Second, the simulation center is a controlled environment, whereas encountering a patient like this one in a real clinical setting could be much more chaotic because learners do not always have defined roles and easily accessible equipment. However, simulation is ideal for a high-acuity, low-frequency case such as tetanus. It enables a safe learning environment for learners to think through the case and identify any gaps in knowledge so that they are

Copyright © 2024 Mangin and Troendle This is an open-access publication distributed under the terms of the Creative Commons Attribution license.

better prepared should a similar situation arise in a real patient encounter.

This specific case may be limited by its complexity. While that complexity is an advantage in some ways, learners missing one thing in the case can lead to a myriad of consequences later on in the simulation. If learners experience difficulty with advancing management, facilitators may need to provide prompts to help them. The students who participated in this simulation were very motivated to learn about tetanus and participate in this challenging simulation. While the case is suitable for highly motivated medical students, its complexity may make it better suited for emergency medicine residents, as many medical students may not see the value in a tetanus simulation. Furthermore, despite rising vaccine hesitancy, tetanus remains rare in the United States and is not likely to be encountered regularly by most learners. However, it is still essential that they understand the unique management and concerns of acute diffuse muscle spasms.

Learners performed significantly better on the posttest than on the pretest. Furthermore, learner feedback was positive. Medical students appreciated the responsibility of caring for a critical patient, the opportunity to perform procedures, and a teamwork approach. Due to this feedback, we are in the process of developing a new case with similar features—muscle rigidity due to strychnine poisoning. Eventually, we would like to run both of these cases consecutively in order to highlight the similarities and differences in presentation and management. We would also like all core rotations to make use of the simulation lab for cases relevant to their individual fields. As medical students often must yield procedures and case management to residents in real-time clinical scenarios, we feel their active engagement in hands-on learning is reflected in their simulation lab performance and posttest scores. The technique of independent learning and solidification of concepts learned in simulation lab can be utilized throughout many fields of medicine.

Appendices

A. Approach to Acid-Base Disturbances.pptx

B. Glycine.pptx

C. Simulation Images and Lab Values.docx

D. Simulation Case.docx

E. Critical Actions Checklist.docx

F. Debriefing Materials.docx

G. Pre- and Posttest.docx

H. Session Evaluation.docx

All appendices are peer reviewed as integral parts of the Original Publication.

Elizabeth Mangin: Third-Year Medical Student, Virginia Commonwealth University School of Medicine; ORCID: https://orcid.org/0009-0003-5986-1948

Michelle Troendle, MD: Associate Professor, Department of Emergency Medicine, Attending Physician, Division of Clinical Toxicology, and Course Director, Critical Care Toxicology for Medical Students, Virginia Commonwealth University Health

Disclosures

None to report.

Funding/Support

None to report.

Ethical Approval

The Virginia Commonwealth University Health Institutional Review Board deemed further review of this project not necessary.

References

1. Wilkins CA, Richter MB, Hobbs WB, Whitcomb M, Bergh N, Carstens J. Occurrence of *Clostridium tetani* in soil and horses. *S Afr Med J.* 1988;73(6):718-720.
2. van Ermengem E. A new anaerobic bacillus and its relation to botulism. *Rev Infect Dis.* 1979;1(4):701-719. https://doi.org/10.1093/clinids/1.4.701. Originally published, in German, in: *Z Hyg Infektionskr.* 1897;26:1-56. https://doi.org/10.1007/BF02220526
3. About tetanus. Centers for Disease Control and Prevention. Updated August 29, 2022. Accessed January 26, 2024. https://www.cdc.gov/tetanus/about/index.html
4. Diphtheria, tetanus, and pertussis vaccine recommendations. Centers for Disease Control and Prevention. Updated March 10, 2023. Accessed January 26, 2024. https://www.cdc.gov/vaccines/vpd/dtap-tdap-td/hcp/recommendations.html
5. National Center for Health Statistics. Health, United States, 2019: Table 031. Centers for Disease Control and Prevention. Updated June 26, 2023. Accessed January 26, 2024. https://www.cdc.gov/nchs/hus/contents2019.htm#Table-031
6. Yaqub O, Castle-Clarke S, Sevdalis N, Chataway J. Attitudes to vaccination: a critical review. *Soc Sci Med.* 2014;112:1-11. https://doi.org/10.1016/j.socscimed.2014.04.018

Copyright © 2024 Mangin and Troendle This is an open-access publication distributed under the terms of the Creative Commons Attribution license.

7. DeSilva MB, Haapala J, Vazquez-Benitez G, et al. Association of the COVID-19 pandemic with routine childhood vaccination rates and proportion up to date with vaccinations across 8 US health systems in the Vaccine Safety Datalink. *JAMA Pediatr*. 2022; 176(1):68-77. https://doi.org/10.1001/jamapediatrics.2021.4251
8. Surveillance. Centers for Disease Control and Prevention. Updated March 7, 2023. Accessed January 26, 2024. https://www.cdc.gov/tetanus/surveillance.html
9. Seither R, Calhoun K, Yusuf OB, et al. Vaccination coverage with selected vaccines and exemption rates among children in kindergarten—United States, 2021–22 school year. *MMWR Morb Mortal Wkly Rep*. 2023;72(2):26-32. https://doi.org/10.15585/mmwr.mm7202a2
10. Khungar V, Poordad F. Hepatic encephalopathy. *Clin Liver Dis*. 2012;16(2):301-320. https://doi.org/10.1016/j.cld.2012.03.009
11. Hurtubise B, MacLellan A. Clinical reasoning: a case of acute encephalopathy and rigidity in a 30-year-old man. *Neurology*. 2019;93(17):759-763. https://doi.org/10.1212/WNL.0000000000008371
12. Wolters EC, Stam FC, Lousberg RJ, et al. Leucoencephalopathy after inhaling "heroin" pyrolysate. *Lancet*. 1982;320(8310): 1233-1237. https://doi.org/10.1016/S0140-6736(82)90101-5
13. Hörtnagl H, Brücke T, Hackl JM. Dysfunction of the sympathetic nervous system in tetanus: a study of 3 cases. *Klin Wochenschr*. 1979;57(8):391-395. https://doi.org/10.1007/BF01480477
14. Berman BD. Neuroleptic malignant syndrome: a review for neurohospitalists. *Neurohospitalist*. 2011;1(1):41-47. https://doi.org/10.1177/1941875210386491
15. Boyer EW, Shannon M. The serotonin syndrome. *N Engl J Med*. 2005;352(11):1112-1120. https://doi.org/10.1056/NEJMra041867
16. Willison HJ, Jacobs BC, van Doorn PA. Guillain-Barré syndrome. *Lancet*. 2016;388(10045):717-727. https://doi.org/10.1016/S0140-6736(16)00339-1
17. Yen LM, Thwaites CL. Tetanus. *Lancet*. 2019;393(10181): 1657-1668. https://doi.org/10.1016/S0140-6736(18)33131-3
18. Farrar JJ, Yen LM, Cook T, et al. Tetanus. *J Neurol Neurosurg Psychiatry*. 2000;69(3):292-301. https://doi.org/10.1136/jnnp.69.3.292
19. Hunter JD, Gregg K, Damani Z. Rhabdomyolysis. *Contin Educ Anaesth Crit Care Pain*. 2006;6(4):141-143. https://doi.org/10.1093/bjaceaccp/mkl027
20. Tiwari TSP, Moro PL, Acosta AM. Tetanus. In: Centers for Disease Control and Prevention. *Epidemiology and Prevention of Vaccine-Preventable Diseases.* 14th ed. Public Health Foundation; 2021:315-328. Updated October 19, 2022. Accessed January 26, 2024. https://www.cdc.gov/vaccines/pubs/pinkbook/tetanus.html#medical

Received: June 5, 2023
Accepted: December 11, 2023
Published: March 29, 2024

Copyright © 2024 Mangin and Troendle This is an open-access publication distributed under the terms of the Creative Commons Attribution license.
